# The association between social connectedness and euthanasia and assisted suicide and related constructs: systematic review

**DOI:** 10.1186/s12889-024-18528-4

**Published:** 2024-04-16

**Authors:** Emma Corcoran, Molly Bird, Rachel Batchelor, Nafiso Ahmed, Rebecca Nowland, Alexandra Pitman

**Affiliations:** 1 UCL Division of Psychiatry, University College London, London, UK; 2 Oxford Centre for Psychological Health, Oxford, UK; 3 North East London NHS Foundation Trust, London, UK; 4 South London & Maudsley NHS Foundation Trust, London, UK; 5 Sussex Partnership NHS Foundation Trust, West Sussex, UK; 6 The Oxford Institute of Clinical Psychology Training and Research, University of Oxford, Oxford, UK; 7 Oxford Health NHS Foundation Trust, Oxfordshire, UK; 8 School of Nursing and Midwifery, University of Central Lancashire, Preston, UK; 9 Camden and Islington NHS Foundation Trust, London, UK

**Keywords:** Loneliness, Social isolation, Social connectedness, Social support, Euthanasia, Assisted suicide, Physician-assisted suicide; systematic review

## Abstract

**Background:**

Euthanasia and assisted suicide (EAS) requests are common in countries where they are legal. Loneliness and social isolation are modifiable risk factors for mental illness and suicidal behaviour and are common in terminal illness. Our objective was to summarise available literature to clarify whether these and related measures of social connectedness might contribute to requests for EAS.

**Methods:**

We conducted a pre-registered (PROSPERO CRD42019160508) systematic review and narrative synthesis of quantitative literature investigating associations between social connectedness and a) requested/actual EAS, b) attitudes towards EAS, and c) a desire for hastened death (DHD) by searching six databases (PsycINFO, MEDLINE, EMBASE, Scopus, Web of Science, Google Scholar) from inception to November 2022, rating eligible peer-reviewed, empirical studies using the QATSO quality assessment tool.

**Results:**

We identified 37 eligible studies that investigated associations with a) requested/actual EAS (*n* = 9), b) attitudes to EAS (*n *= 16), and c) DHD (*n* = 14), with limited overlap, including 17,359 participants. The majority (62%) were rated at medium/high risk of bias. Focussing our narrative synthesis on the more methodologically sound studies, we found no evidence to support an association between different constructs of social connectedness and requested or actual EAS, and very little evidence to support an association with attitudes to EAS or an association with DHD.

**Conclusions:**

Our findings for all age groups are consistent with a those of a previous systematic review focussed on older adults and suggest that poor social connectedness is not a clear risk factor for EAS or for measures more distally related to EAS. However, we acknowledge low study quality in some studies in relation to sampling, unvalidated exposure/outcome measures, cross-sectional design, unadjusted analyses, and multiple testing. Clinical assessment should focus on modifying established risk factors for suicide and EAS, such as hopelessness and depression, as well as improving any distressing aspects of social disconnectedness to improve quality of life.

**Funding:**

UKRI, NIHR.

**Supplementary Information:**

The online version contains supplementary material available at 10.1186/s12889-024-18528-4.

## Background

Euthanasia and assisted suicide (EAS) are becoming legal in an increasing number of countries (including Australia, Belgium, Switzerland, Colombia, and Canada) and US states [1–3], with many other countries debating the issue of legalisation. The UK government is considering whether to allow assisted dying for terminally ill people [4], reflecting a high level of public support but divergent views of politicians, faith groups, and clinicians [5, 6]. Within countries or states where EAS is legal, between 0.3 and 4.6% of deaths are attributed to EAS [3], and the number of EAS requests and deaths attributable to EAS is rising [7, 8]. Most countries operate under strict restrictions, allowing EAS for those with either a terminal illness or serious physical illness causing intolerable suffering that cannot be relieved, such as amyotrophic lateral sclerosis (ALS), or terminal cancer [9, 10]. This is strictly monitored, aiming to achieve the goal that all patients receive the best end-of-life care aligned with their preferences. In some countries (such as Belgium and the Netherlands) EAS is extended to people based on a psychiatric disorder deemed to be causing suffering that is ‘unbearable and without prospect of improvement’, such as treatment-resistant depression [11]; often termed psychiatric EAS [12]. Such cases are often characterised by loneliness or social isolation, and disagreement between clinicians [13], and not all such patients have received evidence-based treatments [14]. The procedures for granting EAS vary substantially between countries and contexts, and decision-making should always take account of modifiable risk factors for suicidal thoughts. There are no reliable figures on how many people travel abroad to access EAS, or the proportion of suicides in which an individual had tried to access EAS. There is also no clear evidence that rates of (non-assisted) suicide change significantly in countries after EAS is introduced [15].

Terminology classifying EAS is contentious, reflecting the strong views of patients, clinicians, and the public regarding the ethics of these practices [16–18]. The term *euthanasia* describes the act of deliberately ending a person’s life to relieve suffering, and can be defined as passive (i.e., by omitting treatment such as life support or life-saving drugs with patient consent) or active (i.e., through deliberately and knowingly administering a sedative or relaxant drug to a patient who does not otherwise need it, at a dose that has the aim of ending their life, with the patient’s consent). The term physician-assisted suicide (PAS) and the related term physician-assisted dying (PAD) apply to cases in which a physician provides a patient with a drug or intervention in the knowledge that the patient will use this to end their life [14, 19, 20]. *Assisted suicide* describes where active euthanasia is enacted by a relative or carer, who has obtained those drugs with the intention of supplying them to an individual, who takes them willingly with the intention of dying. Whilst there is a considerable degree of overlap between the terms euthanasia and assisted suicide, euthanasia is defined as a doctor ending someone’s life with their consent, whereas physician assisted suicide and assisted suicide define the act of supporting someone in ending their life by providing them with the means to do so [21]. Due to these conceptual overlaps, this review uses the unifying label Euthanasia and Assisted Suicide (EAS) to describe euthanasia, PAS, PAD, and assisted suicide.

The term *desire for hastened death* or *desire to hasten death* (DHD) is used among people with a terminal illness to describe suicidal thoughts or requests for assisted suicide or euthanasia, regardless of whether they are legal in the patient’s country [22]. DHD is highly correlated with depression and suicidal ideation and regarded as a marker of profound psychological distress [23], signalling a need to identify and address modifiable risk factors for suicidality. Among people with a terminal illness these include clinical factors, such as pain and depression, and social factors, such as loneliness, poor social support, and financial concerns [24–26]. Reasons for requesting EAS include perceptions of unacceptable quality of life (commonly due to loss of independence, mobility, communication, or participation in meaningful activity) and fear of future suffering or disability [27]. It is therefore important to understand the factors driving people to consider EAS (via DHD), highlighting whether any risk factors can be further modified. This might also inform the development of approaches developed to support people in making an informed decision as to whether to end their life.

Theoretical models of suicide and suicidal behaviour, although they do not apply directly to EAS, propose that suicide arises from a complex combination of biological, social, psychological, and environmental factors, including social connectedness, interacting with adverse life events [28]. Whether a person acts on their suicidal ideation, including whether someone with terminal illness seeks EAS, is likely to be determined by the interplay of these influences. Thwarted belongingness is a key social connectedness construct in theoretical models of suicide that is also likely to apply to decision-making around EAS. It describes what arises when the fundamental need to form and maintain strong, stable interpersonal relationships is unmet, resulting in feelings of disconnection. This is postulated to influence the emergence of suicidal thoughts in someone who feels defeated and entrapped. The Interpersonal Theory of Suicide considers thwarted belongingness as comprising a) loneliness and b) the absence of reciprocal caring relationships [29]. Loneliness is defined as a subjective unpleasant feeling arising from a mismatch between a person’s desired and perceived level of meaningful social relationships [30]. Studies using the approach of confirmatory factor analysis have shown convergent associations of thwarted belongingness with loneliness [31] and there are also correlations of thwarted belongingness with other measures of social connectedness [32]. Loneliness is distinct from more objective measures of social connectedness, such as social isolation, social capital, and social network. Social isolation is an objective measure of the number of social connections, quantified in terms of social network size and number of meaningful ties [33]. It is therefore possible to have a large number of social contacts but still experience feelings of loneliness, or vice versa [34].

Loneliness is associated with a range of adverse health outcomes [35–37], including increased all-cause mortality [38] and suicide [39]. The association between loneliness and suicidal thoughts and attempts [36, 37, 40] is at least partially mediated by depression [40]. Social isolation is also associated with poor physical and mental health [41], and with suicidal thoughts, suicide attempts, and suicide [39, 42, 43]. Conversely, social support protects against suicidal ideation [44]. Given the high prevalence of loneliness and social isolation in people with terminal illness [45], and among people who request EAS for psychiatric indications [13], and its stigmatising nature [45], it is important to understand its contribution to decision-making around EAS. One previous systematic review of factors predicting request for or attitudes to EAS in older adults found no association with loneliness or with satisfaction with family relationships [46]. However, no systematic review to date has summarised associations between social connectedness and EAS across all age groups. In this review we aimed to describe the association between social connectedness and a) requested/actual EAS, b) attitudes to EAS, and c) DHD. We used the umbrella term social connectedness to describe a person’s perceptions of their relationships with others, including constructs such as loneliness, social isolation, social support, and other terms used to describe the quantity and/or quality of relationships (e.g., living alone; satisfaction with relationships). This met our objective to expand our scope to include measures beyond loneliness and social isolation, to capture all studies investigating associations with social disconnectedness.

## Methods

We conducted the review in accordance with Preferred Reporting Items for Systematic Reviews and Meta-Analyses (PRISMA) guidelines [47] (see Appendix 1a and 1b) and guidance for conducting narrative synthesis in healthcare [48]. We pre-registered the review protocol on PROSPERO (CRD42019160508).

### Search strategy

We searched five psychological and medical electronic databases (PsycINFO, MEDLINE, EMBASE, Scopus, and Web of Science) from inception to November 2022. We conducted the initial search on 16th December 2019, repeated this in July 2021 as an interim update, and then finally on 17th November 2022 to capture all papers published until the end of October 2022. We used the following search terms: lonel* OR social isolation* OR social network$ OR social support* OR social contact* OR social relation* OR social capital* OR confiding relation* AND euthan* OR assist* suicide* OR assist* death*. Search terms were agreed by the interdisciplinary team, which included experts in loneliness research and a psychiatrist with experience of psychiatry in palliative care settings. We also reviewed the first 200 hits from Google Scholar for each combination of search terms. Finally, we also used reference searching of potentially eligible articles to identify additional citations.

### Inclusion and exclusion criteria

Our inclusion criteria were any cross-sectional or longitudinal epidemiological study investigating an association between social connectedness (including loneliness, social isolation, social support, social capital, confiding relationships, and thwarted belongingness) and three outcomes a) EAS (requested or actual), b) attitudes towards EAS (including using hypothetical scenarios), and c) DHD. The first of these categories related to our main research question, but we included studies providing information on attitudes to EAS and on DHD to provide a broader perspective.

Studies were included if quantitative data related to routine records of EAS (actual/requested), patients’ ratings (but not solely their caregivers as a proxy measure), or the accounts of involved healthcare professionals or carers. We included studies regardless of whether they used validated measures of the above constructs, and were interested in any effect measures reported.

We excluded studies that were not written in English, not empirical research, and not published in a peer-reviewed journal; excluding books, book chapters, reviews, abstracts, posters, dissertations and editorials. We also excluded studies considering passive euthanasia only, assuming that in such cases a patient would be too incapacitated to reflect on measures of exposure/outcome.

### Screening

One researcher (EC) conducted an initial screen of all titles and abstracts, and a second researcher (MB) conducted an independent screening of a random sample of 20% of citations. Any disagreements were discussed with the wider team to resolve inclusion/exclusion. One researcher (EC) completed a full text screen of all papers, and a second researcher (MB) conducted an independent screen of a randomly selected 20% of full texts. Two other researchers (AP; RB) then conducted independent checks of all included full texts. As with the initial screen, disagreements were discussed within the review team to reach consensus.

### Data extraction

One researcher (EC) extracted from each study details of sample demographics, details of key measures, analytic approach, findings and interpretations, tabulating these separately for each of the three outcomes. Two researchers (AP; RB) reviewed the data extracted for 100% of the studies. Disagreements were discussed within the review team to reach consensus.

### Quality assessment

We conducted quality appraisal of all included studies using an adapted version of the Quality Assessment Tool for Systematic Reviews of Observational Studies (QATSO) [49]. As per a previous systematic review on the topic of loneliness [40] we expanded the original scoring of 0–5 to 0–9, with higher scores reflecting higher quality (Appendix 2). We rated included studies on the quality of design, response rate, whether validated exposure/outcome measures were used, and whether the analysis controlled adequately for potential confounders. Where studies did not state their threshold for statistical significance, we used a threshold of p < 0.05. One researcher (EC) rated quality for all papers. The quality of each paper was independently rated by at least two researchers (EC; MB; AP; RB). Any disagreements over study quality were discussed within the team to reach consensus. No studies were excluded from the review based on quality, but all findings were presented in the context of study quality.

The certainty of the evidence available was assessed using the Grading of Recommendations, Assessment, Development, and Evaluations (GRADE) scoring system [50] with GRADE criteria adapted for a narrative synthesis approach, according to Murad and colleagues [51] (see Appendix 3 for GRADE scoring criteria). Two researchers (AP; NA) independently assessed the certainty of evidence for each research question and met to resolve any inconsistencies.

### Synthesis of findings

We tabulated details extracted from eligible studies, regardless of quality rating, by category of social connectedness measure. We created a table each for our three outcomes: requested/actual EAS; attitudes to EAS; DHD.

We conducted a narrative synthesis of the findings of those studies rated at a low or medium risk of bias, categorised by our three outcomes. We also noted how these findings related to legality of EAS in country/state sampled.

## Results

**Fig. 1:**
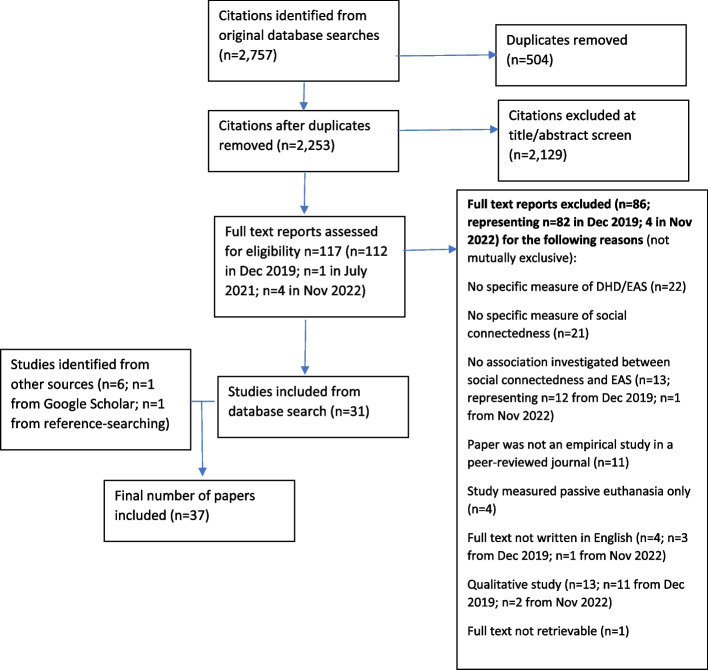
Flow chart of included studies

Our initial database searches identified 2,757 citations, reduced to 2,253 following de-duplication. After title and abstract screening, 112 citations were judged potentially relevant. Following full text screening, we identified 30 studies eligible for inclusion. Google Scholar searches identified a further five eligible studies, resulting in a total of 35 papers. Our July 2021 search identified one further eligible paper, which was included. Our November 2022 search identified four potentially relevant studies, but all were excluded on full text screening. Throughout these searches one relevant study was identified through reference-searching and was included. A total of 37 eligible studies were included in the review (Fig. 1), accounting for 17,359 participants (range 6 to 7,534).

### Characteristics of studies

We categorised the 37 studies by our three outcomes: requested/actual EAS (Table 1; *n* = 9), attitudes to EAS (Table 2; *n* = 16), and DHD (Table 3; *n* = 14), with limited overlap between categories due to use of more than one category of outcome measure. Within each of these tables we sub-classified studies by type of social connectedness exposure measure. We did not conduct a meta-analysis due to heterogeneity of the range of measures and methodologies used in identified studies, as described below.

**Table 1:** Summary of study characteristics and findings for requested/actual EAS (*n* = 9)

**Study/Setting**	**Sample size and characteristics (Total sample size, % female, mean age)**	**Study design, patient population & recruitment procedure**	**Social connectedness measure**	**EAS measure**	**Summary of whether exposure/outcome measures were validated (**✔ or **X**)	**Association between exposure and outcome (**✔ or **X**)	**Statistical analytic approach**	**Main findings**	**Quality assessment and notable methodological limitations**
**Broad measures of social connectedness (n = 2)**									
Smith et al. (2011) [52] USA (Oregon)	N = 149; 43% female; Mean age (SD): 61.0 (13.3) for the group who requested and received PAD 61.2 (12.2) for the group who requested but did not receive PAD 60.1 (14.2) for the comparison group of deceased family members who did not pursue physician assisted death	Cross-sectional survey of the relatives of patients with terminal illness recruited from medical centres, hospices, organisations for patients with Amyotrophic Lateral Sclerosis (ALS), and euthanasia advocacy organisations in Oregon (where physician assisted death has been legal since 1997); 52 who received lethal prescriptions, 34 who requested but did not receive lethal prescriptions, and 63 deceased individuals who did not pursue physician assisted death	Social connectedness measured using 3 items from the Quality of Death and Dying Questionnaire (QODD): time spent with family and friends, attendance at important events, and physical expressions of affection (validated as a full measure but not individual items; note that this was completed by relatives retrospectively rating their relative’s experience)	Request for and actual EAS measured using categorical measures of: death by EAS, request for EAS, or not considering EAS, based on analysis of records kept in hospices, medical centres, and voluntary sector organisations representing those with terminal illness (objective measures based on routine records)	Exposure **X** EAS outcome ✔	**X**	ANCOVA	No significant group differences in scores on the three social connectedness measures between those who died by EAS, requested EAS, or were not considering EAS (effect sizes & p-values not reported)	Medium risk of bias No adjustment for baseline characteristic; control group was a convenience sample of family members recruited from the hospices, medical centres, and the same voluntary sector organisation as the other two groups
Ruijs et al. (2014) [53] Netherlands	N = 76; Mean age 70 52% female Majority were, single and had a middle/lower education. 51% were religious; all patients were Caucasian	Cross-sectional study of all patients on GP lists in Utrecht with terminal cancer who were expected to die within half a year and who were expected to live at home (most of the time) until death, as nominated by GPs	Measures of loneliness, Unsatisfactory social contacts, subjective isolation (capturing a sense of isolation or of ‘no one being present for you’ but termed ‘practical loneliness’); and insufficient support (from family/relatives) from the State-of-Suffering V (SOS-V) [54] measure, developed by the authors as a comprehensive instrument for quantitative and qualitative assessment of unbearable suffering related to 69 physical, psychological, and social symptoms for end-of-life patients, divided into five domains (validated measure)	Explicit request for EAS, as documented by GP (objective measure based on routine records)	Exposure ✔ EAS outcome ✔	**X**	Fisher’s exact test for nominal variables (using Bonferroni correction for multiple testing)	No associations between request for EAS and measures of loneliness, unsatisfactory social contacts, insufficient support, or subjective isolation (effect sizes not reported, p values ranged from p = .420 to p = .995 for social connectedness measures)	Medium risk of bias Whilst overall SOS-V was validated, the 4 measures of social connectedness were of uncertain validity used in isolation
**Social isolation (n = 1)**									
Virik & Glare (2002) [55] Australia	N = 6 patients from a sample of 490 patients referred to the palliative care service over one year; 1 female (age 44) and 5 male (age range 58–78 years) patients Mean age 65	Cross-sectional study of the notes of palliative care patients who had requested euthanasia, as recorded in clinical audit records within a palliative care service	Social isolation measured based on subjective researchers’ judgement (binary measure) as to whether there was little or no contact with or support from family or friends, based on analysis of clinical audit notes (unvalidated measure)	Request for EAS, as documented as a request for euthanasia in the clinical notes (objective measure based on routine records)	Exposure **X** EAS outcome ✔	X	Frequency counts	67% (4/6) patients were judged to be socially isolated; No association described as numbers too small to conduct statistical analysis	High risk of bias No tests of statistical significance used in the analysis of the patient profiles, as the number of patients was too small for any meaningful comparisons
**Loneliness (*n* = 1)**									
Snijdewind et al. (2015) [11] Netherlands	N = 645; Modal age was 80 + ; 62% female; Majority were single	Cross-sectional study of patients requesting EAS in 2013, by analysing content of applications to an end-of-life clinic offering euthanasia or physician-assisted suicide that were concluded during that year	Loneliness measured using frequency counts of mentions of loneliness within application forms (unvalidated measure)	Request for and actual EAS based on records of applications to an end-of-life clinic, categorised based on whether the patient’s request for EAS was: granted (n = 162) rejected (n = 300), withdrawn by the patient (n = 59), or the patient died before a final decision was reached (*n* = 124) (objective measures based on routine records)	Exposure **X** EAS outcome ✔	✔	Frequency counts were compared; bivariate associations of specific variables with EAS application outcome (granted *versus* rejected) presented in table, text lists variables that were independently associated with outcome after being entered into a multivariable regression model (multivariable analysis)	Loneliness was mentioned within the EAS request form for 49% of cases in which EAS was granted *versus* 72% of cases in which it was rejected 213 (p = < .001). Also reported, but did not compare statistically, that loneliness was mentioned in the application forms of 44% of those where the patient died before a decision was made, ad 70% of cases in which the application was withdrawn. Loneliness was independently associated with an EAS application being rejected (O = 0.52; 95% CI = 0.31–0.87; *p* = .01) as was being single, being single, having a solely psychological condition, and having loss of mental capacity. At least 13 other variables were not associated with EAS outcome	Medium risk of bias Exploratory analysis in which loneliness was one of at least 20 variables tested, with the potential for Type I error; analysis described in text as a multivariable regression analysis but no specific confounders listed
**Loss of social role (*n *= 1)**									
Comby & Filbet (2005) [56] France	*N *= 13; mean age 63; range 15-/82 years; 54% female	Cross-sectional survey of palliative care patients requesting EAS from all those in four eligible palliative care units, representing 1.6% of the 611 patients under their care, with measures completed by clinical staff	Loss of social role captured by palliative care staff judgement over whether a patient had experienced loss of social roles e.g., within the family, or within their professional responsibilities because of illness (unvalidated measure)	Request for EAS based on palliative care registry of requests for EAS expressed by terminally ill patients or their families (objective measures based on routine records)	Exposure **X** EAS outcome ✔	**X**	Simple frequency counts; no associations investigated	12/13 patients experienced loss of social roles, and this (along with physical changes and existential suffering) was the most common reason for requesting EAS	High risk of bias highly subjective measure of loss of social role; no comparison with those who did not request EAS; no formal statistical analysis
**Social support (*n *= 4)**									
Ganzini et al. (2006) [57] USA (Oregon) (also see Table 3)	*N* = 161; Mean age 62; 27% female; majority were white and married; mean of 13.5 years in education	Longitudinal study of patients with advanced cancer recruited from oncology clinics in Oregon (where physician assisted death has been legal since 1997), interviewed at baseline in 1998 and followed-up every three months for up to two years	Social support measured using the Duke University of North Carolina Functional Support Questionnaire (FSSQ) (validated measure)	Request for EAS measured as follows; those who indicated they might consider or were planning to request PAS were followed up every three months for up to two years; once referred to hospice, patients were followed monthly, as tolerated. After the patient’s death, each oncologist was asked if the patient had initiated discussion about PAS and any details of discussion (objective measures based on routine records)	Exposure **X** EAS outcome ✔	n/a	Bivariate models to estimate associations at baseline using a proportional odds regression model For 42/161 patients followed up, 9 patient factors were regressed against outcomes	Insufficient power to investigate this outcome (but see Table 3 for DHD). Only 2 patients requested PAS so formal statistical analysis was not possible	High risk of bias analyses not adjusted for potential confounders; small sample size for longitudinal analysis and insufficient power to analyse data for this outcome
Ganzini et al. (2008) [58] USA (Oregon)	N = 83; 43% female; majority white	Cross-sectional survey of family members of deceased patients from the state of Oregon (where physician assisted death has been legal since 1997) who had requested assisted death, recruited from an Amyotrophic Lateral Sclerosis (ALS) support organisation and an assisted suicide advocacy organisation	Perception of the deceased having felt a lack of social support prior to death, as a potential reason for them having requested PAS, rated by family members using a 5 point Likert-type scale (unvalidated measure)	Request for EAS, based on whether patients had considered, requested or died by EAS prior to their death, based on the centralized registry of patients who explicitly requested a lethal prescription from a physician kept at two medical centres in Portland, Oregon (objective measures based on routine records)	Exposure **X** EAS outcome ✔	**X**	Descriptive statistics; ranking of the median score for each factor rated by family members	Family members did not identify social support as an important reason for a PAD request (median = 1/5, IQR = 1.1), and this was ranked at the bottom of the list (alongside depressed mood and financial concerns)	High risk of bias exposure measure highly subjective and subject to recall bias; statistical analysis limited
Ganzini et al. (2009) [59] USA (Oregon)	*N* = 56; Mean age 66; 52% female; majority were white and single/ divorced/ widowed; mean of 16.1 years in education	Cross-sectional survey of people who had requested EAS or contacted a euthanasia information organisation to request advice on PAD	Perception of a lack of social support, rated by patients using a 5 point Likert-type scale (unvalidated measure)	Patient ranking of the importance of their reasons for having requested PAS, rating each between 1 and 5, where 1 was “reason not at all important in decision to request a lethal prescription” and 5 was “reason very important in decision to request a lethal prescription”, in which one option was “Lack of support” (unvalidated measure)	Exposure **X** EAS outcome **X**	**X**	Descriptive statistics ranking the median score for each factor rated by patients, also presenting inter-quartile range	Patients did not identify social support as an important reason for a PAD request (median = 1/5, IQR = 1.1), and this was ranked at the bottom of the list (alongside depressed mood, current mental confusion, and current loss of continence)	High risk of bias exposure measure unvalidated; mixed sample of those who requested EAS formally and those who requested information about it; statistical analysis limited
Smith et al. (2015) [60] USA (Oregon)	*N *= 94; 50% female The majority were married. The ethnicity was mixed	Cross-sectional questionnaire study of patients with terminal illness recruited from end-of-life advocacy organisations and hospices, and nominated by ethics consultants, palliative medicine and oncology physicians at large medical centres	Social support measured using the Duke University of North Carolina Functional Support Questionnaire (FSSQ) (validated measure)	Request for EAS based on having contacted a large end-of-life advocacy organisation for information about accessing PAD, or made an explicit request for PAD to their physician (objective measures based on routine records)	Exposure ✔ EAS outcome ✔	**X**	Student’s t-tests to test for between-group differences on 6 measures (including social support) by PAS request; those significantly correlated at a threshold of p = < 0.20 were entered into a final logistic regression model (multivariable analysis)	No difference in social support for those who requested and did not request PAS (t = -0.67, p = 0.51) and so was not entered into the final logistic regression model	Medium risk of bias possibility of misclassification if those in the control group were in fact intending to pursue PAD but had not yet actioned or articulated it

**Table 2:** Summary of study characteristics and findings for attitudes to EAS (*n* = 16)

**Study/Setting**	**Sample size and characteristics (Total sample size, % female, mean age)**	**Study design, patient population & recruitment procedure**	**Social connectedness measure**	**Attitudes to EAS measure**	**Summary of whether exposure/outcome measures were validate (**✔ or **X**)	**Association between exposure and outcome (**✔ or **X**)	**Statistical analytic approach**	**Main findings**	**Quality assessment and notable methodological limitations**
**Social isolation (*n* = 1)**									
Wilson et al. (2007) [61] Canada	*N* = 379; mean age 67.2 (SD = 12.9); 55% female; majority were white, Protestant, were married, lived with their spouse and had more than a high school education; mean religiosity score was 9.6/15	Cross-sectional semi-structured interview study of patients with terminal cancer recruited from a national palliative care survey	Social isolation measured by summing social network size based on asking patients about number of children, close relatives and friends (unvalidated measure)	Attitude to EAS measured by asking patients if they would ask for EAS if it were legal, as part of a set of standardised questions developed previously by the authors in a previous study [62] (unvalidated measure)	Exposure **X** Attitudes outcome **X**	✔	Chi-square tests and logistic regressions to test associations	Social network size was associated with current interest in EAS I bivariate analyses (OR = 4.0, 95% CI = 1.6–10.5; p = .008)	High risk of bias No adjustment for potential confounders; no correction for multiple testing
**Loneliness (*n* = 1)**									
Buiting et al. (2012) [63] Netherlands	N = 1,245 participants in the Longitudinal Ageing Study Amsterdam for 2008; a study that started in 1992 to recruit a nationally representative sample of people aged 55–84 years. In the sample used for this analysis (aged at least 70), more than half of the respondents were 74 years or younger, 55% were women and about 60% of the respondents were married or had a registered partnership	Longitudinal design, involving repeated cross-sectional surveys of separate samples of older adults to explore changes in attitudes over time. Each was a representative sample of older adults (age 55–85 years), stratified for age, sex, and level of urbanisation	Loneliness measured using the De Jong Gierveld Loneliness Scale (to capture social loneliness and emotional loneliness) (validated measure)	Attitudes to EAS captured by asking patients whether they could imagine themselves ever requesting EAS (unvalidated measure)	Exposure ✔ Attitudes outcome **X**	**X**	Logistic regression using Generalised Estimating Equations (GEE), adjusted for age group and time (multivariable analysis)	No differences in the proportion of people who reported no loneliness (40% vs 41%), loneliness (39% vs 36%). or severe loneliness (21 vs 23%) for people who could imagine requesting EAS vs people who could not image requesting EAS; text reported no significant association of loneliness with desire for EAS but no test statistic presented	Medium risk of bias no test statistic presented for this comparison (the lack of an association reported in text only); those sampled were adults aged over 70 and were not defined by life-limiting illness
**Loneliness and perceived social support (*n* = 1)**									
Cicirelli V (1997) [64] USA (Indiana)	*N* = 388; mean age 73; 74% female; majority were widowed and had some college or vocational training; mean religiosity was 14.14/16; mixed socio-economic status and ethnicity (specifically recruited Black and White older adults)	Cross-sectional survey of older adults aged 60–100 years living in the community in Indiana state, recruited through older adults’ organisations	Loneliness measured using the 20 item UCLA Loneliness Scale (validated measure) Perceived social support measured using the Social Networks in Adult Life Survey to capture the number and closeness of people perceived by an individual to be in his or her support network (validated measure)	Attitudes to EAS using a rating scale of whether EAS was acceptable in 17 different personal situations, each with 7 end-of-life decision options (note that wording was authors’ own): “to strive to maintain life, to refuse medical treatment or request its withdrawal, to allow someone close to decide what is best in the situation, to commit suicide, to ask the doctor (or someone else) for assistance in committing suicide, to ask the doctor (or someone else) to end one's life, and to allow the doctor (or someone else) to decide to terminate life)”, rating each of the seven decision options whether they would or would not decide to do it using a 5-point Likert-type scale from *would not do* (1) to *would do* (5) to indicate their view, then collapsed to form a dichotomous variable indicating if the respondent would be likely to find the option acceptable or not (unvalidated measure)	Exposure ✔ Attitudes outcome **X**	**X**	Factor analysis reduced the 17 outcome variables to three outcomes: Endlife: included the decision options of suicide, assisted suicide, and voluntary euthanasia Others: included the two items in which the end-of-life decision was deferred to a significant other or to the physician Maintain:included the decision options of trying to live as long as possible and refusing or withdrawing treatment (although these were contradictory) Loneliness and perceived social support were tested among 14 different independent variables Hierarchical regression analysis adjusted for ethnicity, gender, socioeconomic status, marital status, age, health, functional dependency, quality of life values, religiosity, fear of death (fear of the dying process, fear of the destruction of the body, fear of the unknown & fear for significant others), wellbeing (self-esteem, depression, life satisfaction & loneliness), locus of control (internal, chance & powerful others), life events, social support (multivariable analysis)	Focussing on the outcome Endlife to represent direct desire for EAS, there was no significant association observed with loneliness (Beta coefficient = .08) or perceived social support (Beta coefficient = -0.02 for inner circle; 0.07 for middle circle; 0.00 for outer circle)	Medium risk of bias unvalidated measure of attitudes to EAS developed by the authors; risk of Type I error given exploratory nature of analysis; older adults sampled were not defined by whether or not they had a life-limiting illness
**Social isolation and thwarted belongingness (*n* = 1)**									
Stolz et al. (2017) [65] Austria	*N* = 493 care-dependent older adults Mean age 74 58% female Majority did not live alone, had primary/ lower secondary education, and considered themselves ‘rather religious’	Cross-sectional survey of care-dependent older adults (age 50 and over) in the community sampled through a multistage random sampling procedure among the general population, with data collection by face-to-face computer-assisted interviews. Care-dependent status was defined as a having a physical or mental disability that was expected to last at least 6 months	Social isolation captured using a binary measure of living alone (objective measure) Thwarted belongingness measured using the thwarted belongingness sub-scale from the Interpersonal Needs Questionnaire (INQ-10) including 5 items such as ‘These days, I feel disconnected from other people’ or ‘These days, I feel like I belong’ (validated measure)	Attitudes towards EAS based on a set of 4 questions asking whether the participant would support EAS in different scenarios (worded as: “‘In case this older, care-dependent person would not want to live on, what of the following would you support?”) to derive four outcomes: Approval of availability of assisted suicide; Hypothetical utilisation of assisted Suicide; Approval of availability of euthanasia; Hypothetical utilisation of Euthanasia (unvalidated measures, based on questions used in previous study by the authors)	Exposure ✔ for both social isolation and for thwarted belongingness Attitudes outcome **X**	✔ social isolation (living alone) **X** thwarted belongingness	Multivariable logistic regression adjusted for gender, age, education, area (village/town/city), religiosity, physical illness (self-rated health, functional limitations, sensory functioning), psychological distress (fear of death, perceived burdensomeness, depressed affect, passive suicide ideation, active suicide ideation), living alone, social trust, trust in doctors (multivariable analysis)	Living alone was associated with approval of availability of euthanasia (OR = 2.54; 1.39–4.63; *p* = < 0.05) and hypothetical utilisation of euthanasia (OR = 3.99; 2.19–7.26; *p* = < 0.05) but was not associated with approval of availability of assisted suicide or hypothetical utilisation of assisted suicide thwarted belongingness was not associated with any of the four outcomes	Low risk of bias
**Satisfaction with social relationships (*n* = 1)**									
Seidlitz et al. (1995) [66] US	*N* = 802 adults **(**541 women and 261 men); Mean age 71 67% female; Majority were white, married, and had religion as a major role in their lives.; mixed socio-economic status	Cross-sectional survey of older adults (aged 60 years and above) capable of independent living, selected through randomly generated telephone numbers via a Gallup telephone survey	Satisfaction with family relationships rated using a 5-point Likert scale (unvalidated measure)	Attitude to EAS captured using one standardised question (used in Gallup surveys for older people) with a 5 point Likert-type scale, alongside four other questions on attitudes to suicide more generally (unvalidated measure)	Exposure **X** Attitudes outcome **X**	**X**	Logistic regression adjusted for age, gender, race, marital status, religiousness, self-rated health, satisfaction with relationships, income (multivariable analysis)	No significant association of satisfaction with family relationships and attitude to EAS; No significant association of satisfaction with family relationships and other attitudes to suicide more generally e.g., that suicide is a personal decision and that others should not get involved; text reported that satisfaction with family relationships emerged as a predictor of the attitude that suicide is a personal decision in the multivariate analyses but p-value was 0**.055** i.e., non- significant	High risk of bias no response rate recorded for telephone sampling; unvalidated measure of satisfaction with family relationships; older adults sampled were not defined by whether or not they had a life-limiting illness
**Social isolation and social support (*n* = 1)**									
Berkman et al. (1999) [67] USA (Oregon, Michigan)	*N* = 511; mean age 50 80% female; majority were white, said religion was very helpful in their lives, were married, and had an undergraduate degree; mixed SES	Cross-sectional survey of patients with Multiple Sclerosis (MS) recruited from the National MS Society (Oregon and Michigan groups), using printed questionnaires (one participant completed on the telephone due to disability)	Social isolation measured by asking about number of close friends and relatives (unvalidated measure) Social support measured using the 19-item MOS-SSS, with items assessing the frequency of availability of social support in the domains of emotional/informational, tangible, affectionate, and positive social interaction (validated measure)	Attitudes to EAS measured using a question asking whether they would consider EAS as an option (yes/no) and rating five hypothetical scenarios when this would be acceptable: if you were experiencing unbearable pain, could no longer do anything that makes your life worth living; were causing a financial burden to caregivers and /or family members; could no longer enjoy anything that makes you happy; were feeling extreme emotional distress Also asked one direct question: *Have you* e*ver thought about* *assisted suicide* *as an option* *yourself?* (unvalidated measures)	Exposure **X** and ✔ Attitudes outcome **X**	**X** for social isolation ✔ for social support	Chi-square tests for bivariate associations with a final multivariable regression model using all variables significantly associated with outcome (multivariable analysis)	Social isolation: People who had ever considered EAS as an option had a significantly lower number of family and friends (5.4; SD = 4.8 *versus* 6.8; SD = 4.9; *p* = < 0.001) in linear test for trend) In the multivariable regression model social isolation not significantly associated with positive attitude to EAS in any of the hypothetical scenarios, or to whether they had ever considered EAS Social support: People who had ever considered EAS as an option had a significantly lower score on social support scale (47.6 points, SD = 19.6 *versus* 56.8, SD = 16.9; *p* = < 0.001 using Pearson chi-square test) In the multivariable regression model higher scores on the Social Support Scale were associated with a lower likelihood of considering assisted suicide for all of the hypothetical scenarios (OR for experiencing unbearable pain = 0.98; 95% CI = 0.97,0.99; *p* = < 0.01; OR for no longer enjoying anything that makes life worth living = 0.99; 95% CI = 0.97, 0.99; *p* = < = 0.05; OR for could no longer do anything that makes one happy = 0.99; 95% CI = 0.97, 0.99; *p* = < = 0.05; OR for feeling extreme emotional distress = 0.97; 95% CI = 0.96, 0.99; *p* = < = 0.001) except for causing financial burden (non-significant). Note that two of these reported p values were of borderline significance Higher scores on the Social Support Scale were significantly negatively associated with having ever thought about assisted suicide (OR 0.98; 95% CI = 0.97, 0.99; p = < = 0.05) but again this reported p value was of borderline significance	Medium risk of bias membership of MS Society not representative of all patients with MS; specific confounders unclear
**Social support (*n* = 10)**									
Achille & Ogloff (2004) [68] Canada, UK, & US	*N* = 44; mean age 52; 25% female Majority were Protestant, attended weekly services and prayed daily. Majority were married and lived with spouse, and had a college/university education	Cross-sectional survey of patients with Amyotrophic Lateral Sclerosis (ALS) recruited via advertisements in an ALS email newsletter sent weekly to a reported readership of 3,000 worldwide	Social support measured using the Social Support Questionnaire (SSQ-6) with 6 items capturing network availability and perceived satisfaction with the support available (validated measure)	Attitudes to EAS measured using Likert-type scales asking if participants could see themselves asking for EAS in the future, or whether they would have already asked for this if it were legal (unvalidated measures)	Exposure ✔ Attitudes outcome **X**	✔	Correlation	Negative correlation between availability of social support and whether patients were contemplating EAS (*r* = -.34, *p* < .05)	Medium risk of bias presented simple correlations rather than a multivariable model
Arnold (2004) [69] USA	*N* = 148; mean age 60; 57% female; majority were white and lived alone	Cross-sectional survey of patients with terminal illness recruited via national and state membership newsletters by a right-to-die organisation and local newspapers in a south-eastern USA state	Perceived social support from family, friends and significant others measured using the 12-item Multidimensional Scale of Perceived Social Support (MSPSS) (validated measure)	Attitude to EAS captured by asking if patient had considered hastening their death in the future (yes/no) (unvalidated measure)	Exposure ✔ Attitudes outcome **X**	✔	Discriminant function analysis; adjusted for depression, anxiety, pain, social support, hope (multivariable analysis)	Unadjusted effect size: Wilks lambda = .736 (p-value not reported) Adjusted effect size: Wilks lambda = .79 (*p* = .001)	High risk of bias Recruitment was via those who were members of a right-to-die organisation
Blank et al. (2001) [70] USA (Connecticut)	*N* = 158; mean age 74; 62% female; majority were white and unmarried	Cross-sectional study of older adults surveyed in an acute general medical hospital who had a life expectancy of over 6 months	Perceived social support measured using rating scales of perceived instrumental (i.e., practical), emotional, and financial support (standardised questions taken from the Established Populations for Epidemiologic Studies of the Elderly, but unvalidated measures)	Attitudes to EAS measured using responses to a range of hypothetical scenarios in which they might accept or refuse PAS: if they recovered after illness, if they had limited mobility after illness, if they had cognitive impairment, if they had a terminal illness, and if they required nursing home care. Also asked to rate their responses if they were under financial constraints (unvalidated measure)	Exposure **X** Attitudes outcome **X**	**X**	t-tests; chi-square tests; final logistic regression model including all those variables significantly associated with positive attitudes to PAS, including depression, marital status, religious coping, social support (multivariable analysis)	Social support was significantly associated with considering accepting PAS for only one of the hypothetical scenarios (recovering from illness and being restored to current condition; t = .199; *p*-value < .05) but not for those scenarios in which they had a imagine they had a terminal illness No significant association found when entered into multivariable model Adjusted effect size OR = 0.69; 95% CI = 0.34–1.43; *p*-value not shown) Those with lower social support were more likely to change their preferences and show more interest in PAS if in a situation of financial constraints (*p* = < 0.05)	High risk of bias Unvalidated measures; complex hypothetical scenarios to interpret where measuring outcome; no correction for multiple testing; older adults sampled were not defined by whether or not they had a life-limiting illness
Breitbart et al. (1996) [71] USA (New York)	*N* = 378; mean age 39; 23% female; mean education level 13 years; mixed ethnicity	Cross-sectional study of ambulatory HIV-infected patients recruited from three New York hospitals and other sites serving HIV patients as part of a questionnaire study of pain in patients with AIDS	Perceived adequacy of social support measured using the Social Support Questionnaire (SSQ), capturing two dimensions—number of social supports; quality of social supports (validated measure)	Attitudes to EAS measured using questions asking whether participants would consider EAS, using standardised questions from a previous questionnaire study (unvalidated measure)	Exposure ✔ Attitudes outcome **X**	✔	t-tests; discriminant function analysis, adjusted for death of friend/family member, suicidal ideation, race/ethnicity, quality of social supports, frequency of attending religious services (multivariable analysis)	Significant association between number of social supports and attitude to EAS ( t = -2.41; p = 0.02) Significant association between quality of social supports and attitude to EAS (t = -2.66; *p* = 0.009) The final multivariable model, developed using discriminant function analysis, included quality of social supports, and correctly predicted 60% of cases (*p* = < .0001)	Medium risk of bias not a representative sample of patients with HIV; attitudes to EAS in the mid-1990s will be influenced by public understanding of HIV prognosis at the time of sampling
Emanuel et al. (2000) [13] US (four randomly selected sites nationally)	*N* = 988; mean age 67 52% female Majority were white, Protestant, somewhat/ not religious, married living with their spouse and had graduated from high school. The SES was mixed	Longitudinal (prospective cohort) study of patients with terminal illness referred by their physicians and their primary caregivers and interviewed at two points (T0 = at recruitment, T1 = 2–6 months after the baseline interview)	Social support measured using the 19-item MOS-SSS, with items assessing the frequency of availability of social support in the domains of emotional/informational, tangible, affectionate, and positive social interaction (validated measure) Additional descriptive measure: Caregivers were asked whether they perceived they had adequate social support, but measure/wording not stated (unvalidated measure)	Attitudes towards EAS and personal interest in EAS measured at baseline and follow-up by asking patients a set of standardised questions about their views on EAS used in previous surveys (e.g., “When a person has a disease that cannot be cured, do you think doctors should be allowed by law to end a patient’s life by some painless means if a patient and his family request it?”) and whether they would consider EAS in a hypothetical situation of pain, and of feeling a burden on others, in the context of terminal illness. Also asked questions about whether they had ever considered EAS (unvalidated measures but some standardised questions on attitudes to EAS) Additional exposure measure (ineligible for inclusion in study but reported here): Family members /caregivers were also asked whether the patient had discussed EAS or hoarded drugs (unvalidated measures but some standardised questions on attitudes to EAS)	Exposure ✔ Attitudes outcome **X**	**X**	Multivariate regression models testing variables in groups, then selecting variables that were significantly associated in groups, then conducting a final stepwise regression (multivariable analysis)	No difference in overall social support for patients who did/did not discuss EAS or hoard drugs (*p* = .71) [Caregivers who perceived adequate social support were significantly more likely not to express support for EAS (OR = 0.63; 95% CI = 0.44–0.90)]	Medium risk of bias Sampling bias as physicians/carers were asked to suggest patients; small numbers for statistical analysis involving social support variable
Ganzini et al. (1998) [72] USA (Oregon; Washington)	*N* = 100; Mean age 54 39% female; majority were white and married. They had a mean of 14.4 years of education, and the mean religiosity score was 73.8/100	Cross-sectional questionnaire survey of patients with Amyotrophic Lateral Sclerosis (ALS) and their caregivers recruited from the ALS clinic at Oregon Health Sciences University in Portland, who had participated in previous studies of ALS at the university or had expressed an interest in participating in research on ALS in a prior survey conducted in 1996 on behalf of the university by the Muscular Dystrophy Associations of Oregon and Washington states	Social support measured using the Duke University of North Carolina Functional Support Questionnaire (FSSQ) (validated measure)	Attitudes towards EAS among patients measured using a set of questions asking whether they would ever consider taking a drug to end their life (unvalidated measures) Additional descriptive measure: Attitudes towards EAS among caregivers measured using questions on whether they would support or oppose the patient’s decision to take a drug to end their life, and the perceived likelihood that the patient would request such a prescription (unvalidated measures)	Exposure ✔ Attitudes outcome **X**	**X**	Chi-square tests for categorical variables, t-tests for continuous variables	Social support did not differ significantly between patients who would consider EAS and those who would not (finding provided in text but not tables as only the significant associations were tabulated)	Medium risk of bias unvalidated measure of attitudes to EAS; unclear whether group differences on social support were analysed in relation to social support as a continuous or categorical variable
Himchak (1997) [73] USA (New Jersey, New York, Pennsylvania)	*N* = 329; mean age 74 75% female The majority were white, Catholic, widowed and lived alone. The SES was mixed	Cross-sectional survey of older adults living in three US states (New Jersey, New York, Pennsylvania) recruited (unclear how) from a range of settings: 216 individuals recruited from group settings such as supported housing, lunch clubs, and senior social clubs, and 113 individuals who were more isolated due to being homebound or not participating in community projects	Social support measured using the Social and Family Contact Scale to capture two dimensions: social support experienced in relationships and social support experienced through decision-making, with categories addressing family structure, frequency of interaction, and mutual aid and assistance. (apparently unvalidated measure; unable to find further information on this measure) Social support also measured using the Family Contact Index, a 4-item scale measuring frequency of contacts for each friend/relative, including those living with them (apparently unvalidated measure; unable to find further information on this measure)	Attitudes towards EAS measured using nine questions taken from five national polls (e.g., Gallup), wording not provided, but using a 3 point Likert-type scale to rate agreement (standardised but unvalidated measures)	Exposure **X** Attitudes outcome **X**	**X**	ANCOVA, correlation, and multiple regression analysis (multivariable analysis)	ANCOCA showed that social support was not associated with attitudes to EAS (values not reported) Social support was not correlated with attitudes to EAS (Correlation coefficients for four dimensions of social support ranged from *r* = -0.08 to 0.02, *p* = 0.16 to 0.67) Multiple regression found that social support alone did not explain a significant portion of the 11% variance in attitudes to EAS contributed by all variables in the model Were association, values not reported ANCOVA No significant association, values not reported	Medium risk of bias unclear how sample recruited, sample were older adults regardless of life-limiting illness; no response rate stated
Lulé et al. (2014) [74] Germany (also see Table 3)	*N* = 93; Mean age 59; 58% female; majority were married; mean of 10.98 years in education	Longitudinal study of patients with Amyotrophic Lateral Sclerosis (ALS) recruited at an outpatient neurology clinic, interviewed at 3 timepoints: T1 baseline interview, T2 6 months, T3 12 months	Perceived social support measured using the emotional scale of the 14-item German version of the Social Support Questionnaire (F-SozU K-14) (validated for a German sample)	Attitudes towards EAS measured using two questions about behaviour/attitudes to end-of-life intervention: “Did you seek information how to shorten life?’’ and ‘‘Should euthanasia be allowed? ‘‘ (response options ‘‘yes’’ or ‘‘no’’) (unvalidated measures)	Exposure ✔ Attitudes outcome **X**	**X**	Repeated measures ANOVAs were conducted separately for all dependent and independent variables (including DHD, attitudes to EAS and perceived social support), providing comparisons at each of the three timepoints Kruskal–Wallis ANOVA to estimate associations of six factors and attitudes to life-prolonging treatment multivariate logistic regression to examine which variables predicted desire for hastened death, mutually adjusted for quality of life, depression, feeling of being a burden, physical function, age and perceived social support	No association of perceived social support with attitudes to EAS (but no test statistics reported)	Medium risk of bias unvalidated measures of attitudes to EAS
Marrie et al. (2017) [75] US (nationwide)	*N* = 7,534; Mean age 60; 80% female; majority were white and had an undergraduate degree	Cross-sectional survey of patients recruited from a national Multiple Sclerosis (MS) research register (the North American Research Committee on MS)	Perceived social support measured using the 5-item Modified Social Support Survey (MSSS), using the items from the 18-item MSSS that are most strongly correlated with the total score rated using a Likert-type scale (validated measure)	Attitudes towards EAS measured by patients rating the degree to which they would consider assisted suicide in five hypothetical scenarios (unbearable pain, financial burden to caregivers, extreme emotional distress, inability to do things that make you happy, inability to enjoy anything) using the options: definitely would consider, probably would consider, probably would not consider, definitely would not consider (as used in a previous study but unvalidated measures)	Exposure ✔ Attitudes outcome **X**	**X**	Logistic regression adjusted for sex, race, age, annual income, disease duration, disability, depression, anxiety, pain, social support, religion, use of an immune therapy (multivariable analysis)	Bivariate association of social support with having positive attitudes to PAS (*p* = < 0.0001); multivariable logistic regression found no association when other variables were adjusted for (adjusted OR = 0.82; 95% CI = 0.59–1.13) but reported as significant in the text. In a supplementary analysis, presented in online e-tables, low social support was not associated with “definitely considering physician-assisted death in all situations”, under any of the five hypothetical scenarios, in fully adjusted models. In a set of further supplementary analyses, there was a significant association between low social support and definitely or probably considering PAD in a hypothetical situation of unbearable pain, but it was not clear what covariates were adjusted for in these four sets of analyses for five different hypothetical situations	Medium risk of bias Unvalidated measure of attitudes to EAS; contradictory findings reported in text and tables; unclear whether the same covariates were used in all models; multiple analyses were presented in supplementary tables with high risk of Type I error)
Pacheco et al. (2003) [76] USA (Ohio)	N = 24 with baseline and follow-up data (of 38 at baseline). Mean age 60 0% female; majority were married and very/ moderately religious; mixed ethnicity	Longitudinal study of patients with terminal cancer recruited from an oncology clinic; T0 questionnaire upon enrolment, T1 three months later (or upon significant decline in health status), and every six months thereafter (or upon significant decline in health status), up to a maximum of five follow-up measures	Social support measured using two dimensions from the 15 dimensions of the Coping Orientations to Problems Experienced Scale (COPE): seeking (i.e., use of) social support for instrumental (practical) reasons, and seeking (i.e., use of) social support for emotional reasons (validated measure)	Attitudes towards EAS, measured using five questions (covering PAS legalization, Euthanasia legalization, PAS request, and Euthanasia request) with agreement captured using a 5-point Likert-type scale (unvalidated measure)	Exposure ✔ Attitudes outcome **X**	**X**	Biserial correlations to examine whether change in attitudes towards EAS was related to change in coping strategies	Use of support for emotional reasons was the only COPE dimension that increased significantly over follow-up (*p* = 0.04), but attitudes to EAS did not change significantly over follow-up, suggesting no association. No cross-sectional associations were tested The correlations between attitudes towards EAS and change in seeking social support for emotional reasons were negative (i.e., those who changed to or stayed with conservative views increased more in use of social support for emotional reasons as compared to the increase in those who changed to or stayed liberal), although significance levels were not stated The biserial correlations between attitudes towards EAS legislation and seeking social support for emotional reasons were significant (PAS legalization -.50; E legalization -.49), while the correlations between attitudes towards EAS request and seeking social support for emotional reasons were moderate (-.34 and -.37, respectively), but not significant	High risk of bias Small sample size; unclear whether all patients were invited to participate; unvalidated outcome measure; comparisons based on correlations

**Table 3:** Summary of study characteristics and findings for DHD (*n* = 14)

**Study/Setting**	**Sample size and characteristics (Total sample size, % female, mean age)**	**Study design, patient population & recruitment procedure**	**Social connectedness measure**	**DHD measure**	**Summary of whether exposure/outcome measures were validated (**✔ or **X**)	**Association between exposure and outcome (**✔ or **X**)	**Statistical analytic approach**	**Main findings**	**Quality assessment and notable methodological limitations**
**Loneliness (*n* = 1)**									
Stutzki et al. (2014) [77] Germany/ Switzerland	N = 66; Mean age 62 41% female Majority were Catholic, married, and had lower secondary education	Longitudinal study of patients with Amyotrophic Lateral Sclerosis (ALS) recruited from ALS clinics and their caregivers, surveyed at two points: T0 conducted as soon as the patients had been informed about the option of life-sustaining measures, and T1 conducted when the patients’ scores on the Amyotrophic Lateral Sclerosis Functional Rating Scale (ALSFRS) had deteriorated by > = 5 points (but not later than 15 months after baseline; average 13.2 months)	Loneliness measured using a question developed for the purpose of this study, capturing feelings of loneliness using a 10-point rating scale (unvalidated measure)	Desire for hastened death (DHD) measured using a 10-point Likert-type scale asking how strong the patient’s current wish was to hasten their death (face validity established only) Also captured descriptive measure of caregivers’ views on whether they could imagine helping the patient to hasten death by means of suicide assistance or euthanasia (yes/no) (face validity established only)	Exposure **X** DHD outcome **X**	✔	Generalised linear mixed regression models with no adjustment for confounders	Loneliness was significantly associated with desire to hasten death (OR = 1.20, 95% CI = 1.02–1.38; *p* = .021), and text mentioned that this was the case at both time points	High risk of bias some recruitment via caregivers, no adjustment for confounders or for multiple testing, unvalidated measures
**Satisfaction with social relationships (*n* = 1)**									
Cheung et al. 2020 [78] (New Zealand) NB: study conducted before EAS became legal in NZ	*N* = 771 palliative care patients; mean age 76.0 years (SD 11.6; range 20–100). About half (50.1%) of the sample were female, and most (87.0%) were European (Maori 8.7%, Asian 1.7%, Pacific people 1.7%, and other 0.9%)	Cross-sectional study of palliative care patients who had received a Resident Assessment Instrument for Palliative Care (RAI-Palliative Care) needs assessment tool anywhere in New Zealand between January 1 and December 31, 2018	Satisfaction with family relationships rated by researchers using multiple sources of information (e.g., referral note, face-to-face interview, observation, discussion with family, carers, or health professionals) to rate degree to which patient had a strong and supportive relationship with family (unvalidated measure)	Desire to hasten death (DHD) captured using responses to an item on the RAI-Palliative Care questionnaire exploring the person’s ‘wish of wanting to die now’, with responses coded as Yes/No/Unable to determine, as derived from interview with the patient and family about advanced directives and end-of-life wishes (9.3% of the sample responded yes, 59.8% reported no, and for 30.9% assessors were unable to determine a response) (unvalidated measure)	Exposure **X** DHD outcome **X**	**X**	Bivariate associations of individual characteristic with wanting to died now; only performed logistic regression on those variables that were significant on chi-square testing	No association between measure of strong and supportive relationship with family and wanting to die now (on Chi2 testing; not entered into multivariable model)	High risk of bias unvalidated measure of satisfaction with family relationships relying on multiple sources; no adjustment for multiple testing
**Social support (*n* = 24)**									
Breitbart et al. (2000) [22] USA (New York)	*N* = 92; Mean age 66; 60% female; majority were Catholic, separated and had more than a high school education; mixed ethnicities	Cross-sectional survey of patients with terminal cancer in a palliative care hospital providing responses via interviews	Social support measured using the Duke University of North Carolina Functional Support Questionnaire (FSSQ) (validated measure)	Desire for hastened death (DHD) measured using the Schedule of Attitudes Towards Hastened Death (SAHD) (validated measure)	Exposure ✔ DHD outcome ✔	**X**	Correlational analysis; stepwise multiple regression analysis to identify variables predicting DHD (multivariable analysis)	Social support was not correlated with desire for hastened death. The final multiple regression model was significantly associated with DHD (overall F = 18.79; *p* = < 0.001) with the inclusion of social support (partial F = 4.35; *p* = 0.05; note borderline significance), as well as hopelessness, depression, and overall physical functioning There was no significant association between perceived quality of social support and DHD (*p* = 0.64)	Low risk of bias
Chochinov et al. (1995) [79] Canada	N = 199; mean age 71; 52% female;majority were Protestant, married, living with family and friends, and had less than a high school education	Cross-sectional survey of patients with terminal illness in hospital palliative care units, using diagnostic interviews and self-reported scales (mainly administered orally)	Perceived social support (quantity and perceived quality) measured by asking patients to indicate the number of family members and friends they have weekly contact with and rate the supportiveness (perceived support score) of these contacts out of 100 using a visual analogue scale (unvalidated measures) Patients also rated the perceived supportiveness of the nursing staff out of 100 (unvalidated measure) (unvalidated measures)	Desire for hastened death (DHD) measured using a set of questions created by the authors for this study, now termed the Desire for Death Rating Scale (DDRS), and since used widely as a set of standardised questions, including by other authors represented in this review, but not formally validated) (unvalidated measure)	Exposure **X** DHD outcome** X**	**X**	Correlations followed by t-tests and chi-square tests for those variables shown to be correlated with DHD; then used a stepwise multiple logistic regression procedure to identify the conjoint predictive value of the individual variables found to be associated with DHD (multivariable analysis)	No correlation of quantity of family and friend support with DHD, or of perceived quality of family and friend support with DHD (reported to be of marginal significance but *p* = 0.06) t-tests showed no association of family weekly contact with DHD, or of friends weekly contact with DHD, but a significant association of quality of family support with DHD (*p* = 0.01) and no association of quality of friend support with DHD t = -.25; *p*-value = 0.8 (family weekly contact) t = .32; *p*-value = 0.75 (friends weekly contact) t = 2.57; *p*-value = 0 .01 (family support score) t = 0.92; p-value = 0.36 (friends support score) Three variables reported to be significant correlates of the DHD (depression, pain, and quality of family support) were entered into the multiple logistic regression model, but only depression was a significant predictor of desire for death, and was significantly collinear with family support (*r *= -0.25, *N* = 196, *p* < O.OO1). Family support did not make a unique contribution to the model	Medium risk of bias used unvalidated measures of exposure and outcome, no correction for multiple testing
Ganzini et al. (2006) [57] USA (Oregon) (also see Table 1)	*N* = 161; Mean age 62; 27% female; majority were white and married; mean of 13.5 years in education	Longitudinal study of patients with advanced cancer recruited from oncology clinics in Oregon (where physician assisted death has been legal since 1997), interviewed at baseline in 1998 and followed-up every three months for up to two years	Social support measured using the Duke University of North Carolina Functional Support Questionnaire (FSSQ) (validated measure)	Desire for hastened death (DHD) measured using Likert-style scale rating i) the degree to which they had considered requesting a legal lethal prescription in the previous two weeks (main outcome), ii) their desire for death to come sooner in the previous two weeks, used as an ordinal scale (unvalidated measures) Insufficient power to investigate the following outcome: Objective request for EAS measured as follows; those who indicated they might consider or were planning to request PAS were followed up every three months for up to two years; once referred to hospice, patients were followed monthly, as tolerated; after the patient’s death, each oncologist was asked if the patient had initiated discussion about PAS and any details of discussion (objective measures based on routine records)	Exposure ✔ DHD outcome** X**	**X **(association between increasing social support and increasing interest in obtaining a lethal prescription, but likely to be a spurious association in view of small sample size)	Bivariate models to estimate associations at baseline using a proportional odds regression model For 42/161 patients followed, 9 patient factors were regressed against whether interest in obtaining a lethal prescription (ordinal outcome) changed over time, using a random effects model	Social support was not associated with likelihood of considering requesting a lethal prescription at baseline (OR = 0.96, 95% CI = 0.92–1.00; *p* = .06) in a bivariate model; only three variables (hopelessness, importance of religion, and quality of life) were entered into the final multivariable model For the 42 subjects followed up longitudinally, increasing social support was associated with increasing interest in obtaining a lethal prescription (coefficient = -0.119; SE = 0.037; *p* = 0.002), as was declining functional status, increasing depression, increasing hopelessness, increasing sense of burden to family, increasing degree to which poor health limited quality of life, increasing suffering Only 2 patients requested PAS so formal statistical analysis was not possible	High risk of bias small sample size for longitudinal analysis; association with social support not adjusted for potential confounders; findings on social support in longitudinal analysis seem anomalous particularly in the context of other variables identified reflecting negative experiences
Kelly et al. (2003) [80] Australia	*N* = 256; Mean age 66 48% female; majority were Protestant, married, and living with their spouse	Cross-sectional survey of patients with terminal cancer (completed with the assistance of a carer if required), recruited from hospices and palliative care services in Brisbane from 1998–2001	Social support measured using the Social Support Questionnaire, 12-item measure capturing both the total number of social supports, and satisfaction with social support (validated measure) Perceptions of family interaction using the Family Relationships Index (FRI) to capture family cohesion (validated measure)	Desire for hastened death (DHD) measured using a modified version of the Desire for Death Rating Scale (DDRS), a set of standardised questions developed by Chochinov et al. 1995 [79] (unvalidated measure) In the current study the authors substituted one item on whether they had discussed this wish to die with two items in order to specify whether it was with family/friends or with a health professional	Exposure✔ DHD outcome **X**	✔	Discriminant function analysis used to predict membership of the group expressing DHD	Discriminant function analysis identified that the following variables were significantly associated with DHD: lower family cohesion scores (on FRI), lower number of social supports, less satisfaction with social supports Standardised correlation coefficients were: -0.29 (family cohesion) -0.13 (number of social supports), -0.10 (satisfaction with social supports) Within-group correlation coefficients were: -0.32 (family cohesion)-0.21 (number of social supports), -0.40 (satisfaction with social supports) Other variables also showing a significant association included: higher depression scores, hospice treatment setting, greater perceived burden on others, higher anxiety, and higher physical symptom scores	Low risk of bias some risk of social desirability bias where carers had assisted patients
Lulé et al. (2014) [74] Germany (also see Table 2)	*N* = 93; Mean age 59; 58% female; majority were married; mean of 10.98 years in education	Longitudinal study of patients with Amyotrophic Lateral Sclerosis (ALS) recruited at an outpatient neurology clinic, interviewed at 3 timepoints: T1 baseline interview, T2 6 months, T3 12 months	Perceived social support measured using the emotional scale of the 14-item German version of the Social Support Questionnaire (F-SozU K-14) (validated for a German sample)	Desire for hastened death (DHD) measured using the Schedule of Attitudes towards Hastened Death (SAHD) (validated measure)	Exposure ✔ DHD outcome ✔	**X**	Repeated measures ANOVAs were conducted for comparisons of all measures at each of the three timepoints and for both outcomes (DHD and attitudes to EAS); Kruskal–Wallis ANOVA to estimate associations of six factors and attitudes to life-prolonging treatments; multivariate logistic regression to examine which variables predicted desire for hastened death, mutually adjusted for quality of life, depression, feeling of being a burden, physical function, age and perceived social support	No association of perceived social support with desire for hastened death (but no test statistics reported)	Medium risk of bias unvalidated measures of attitudes to EAS
O’Mahony et al. (2005) [23] USA (New York) (same sample as O’Mahony et al. (2010) below)	*N* = 64; mean age 54; 52% female; majority were married and had a college education; ethnicity was mixed	Longitudinal (prospective observational) study of patients with terminal cancer recruited from cancer hospitals and palliative care services, T0 baseline and T1 four weeks later (± one week)	Perceived social support measured using the Bottomley Cancer Social Support Scale (BCSSS) at baseline and follow-up; a 9-item, cancer-specific, social support scale assessing perceived adequacy of social support in patients with cancer (validated measure) also measured living alone at baseline (objective measure)	Desire for hastened death (DHD) measured using a modified version of the Desire for Death Rating Scale (DDRS), a set of standardised questions developed by Chochinov et al. 1995) [79] (unvalidated measure) In the current study the authors agreed on their own cut-offs	Exposure ✔ DHD outcome **X**	✔ for perceived social support ✔ for living alone	Linear regression was planned to measure the association between variables and desire for death, but social support was not entered into the model; only chi-square tests and correlation analyses were reported for social support variable	Low social support moderately correlated with desire to hasten death at baseline (*r* = 0.38, p < .01); text indicated that social support scores were higher for those without DHD, and that social support score categories (decreasing, stable, increasing) differed significantly by DHD trajectory but these chi-square tests for changes in social support were not interpretable as direction not stated; DHD scores increased significantly over follow-up (*p* = 0.03) but social support scores did not change significantly (p-0.051), although the association was not formally tested DHD at both baseline and follow-up was associated with living alone (chi-square = 5.98, *P* = < 0*.*02)	Medium risk of bias Only 53 patients completed the social support measure at baseline; unvalidated measure of DHD
O’Mahony et al. (2010) [81] USA (New York) (same sample as O’Mahony et al. (2005) above, but linked to mortality data)	*N* = 64; Mean age 54;52% female	Longitudinal (prospective observational) study of patients with terminal cancer recruited from cancer hospitals and palliative care services, linked to mortality data for survival analysis (analysed data from above study by O’Mahony et al. (2005) but linked data to mortality outcomes)	Perceived social support measured using the Bottomley Cancer Social Support Scale (BCSSS) at baseline and at 4 week follow-up; a nine-item, cancer-specific, social support scale assessing perceived adequacy of social support in patients with cancer (validated measure)	Desire for hastened death (DHD) measured at baseline and at 4 week follow-up using a modified version of the Desire for Death Rating Scale (DDRS), a set of standardised questions developed by Chochinov et al. 1995 [79] (unvalidated measure) In the current study the authors agreed on their own cut-offs	Exposure ✔ DHD outcome **X**	✔	Generalized linear model with DHD as dependent variable and the other variables as predictors (multivariable analysis)	Social support was associated with DHD in adjusted (but not unadjusted) analyses Unadjusted effect size *r* = 0.25; *p* = 0.068 Adjusted effect size B = 0.496; *p* = 0.004 Over follow-up, mean DHD was not significantly lower for persons with improvement in perceived social support versus worsened social support (1.13 vs. 0.77, *p* = .39) In a model including depression, pain, social support, physical functioning, and existential well-being, two variables (physical functioning and existential well-being) were associated with lower group DHD. When an interaction term was included in this model between social support and physical functioning, lower perceived social support was significantly associated with DHD (β = 0.496, *p* = .004), meaning that the relationship between perceived social support and DHD was related to physical performance status [lower social support was also associated with shorter survival time, which was the primary research question]	Medium risk of bias Small sample size (as above); unvalidated measure of DHD
Rodin et al. (2007) [82] Canada (Toronto)	*N* = 326; median age 61.9; 43% female	Cross-sectional study of patients with metastatic cancer recruited from consecutive patients attending their outpatient medical and/or radiation oncology clinic appointments with a treating oncologist at a network of acute care cancer centres in Toronto	Social support measured using the 20-item MOS-SSS, with items capturing 1) emotional/informational support; 2) tangible support; 3) affectionate support; 4) positive social interactions; and 5) global social support (validated measure)	Desire for hastened death (DHD) captured using the Schedule of Attitudes toward Hastened Death (SAHD) (validated measure)	Exposure ✔ DHD outcome ✔	**X**	Stepwise, backward elimination regressions analysis was used to build a multivariate model using socio- demographic variables, physical and illness-related factors, and psychosocial and psychological variables Repeated in the subset of 251 patients who had died by the time of the analysis	Once all variables were entered into the model, social support did not contribute to an association with DHD in either the full sample or the subset who had died. Hopelessness, depression and poor physical functioning were the main predictors (but not physical functioning for the subset model) Overall, 79% reported good social support	Low risk of bias
Rosenfeld et al. (2000) [83] USA (New York State)	*N* = 92; mean age 66; 60% female; majority Catholic; mean years of education was 12.7 years; mixed ethnicity	Cross-sectional interview study of patients with terminal cancer recruited from a palliative care hospital	Social support measured using the Duke University of North Carolina Functional Support Questionnaire (FSSQ) (validated measure)	Desire for hastened death (DHD) captured using the Schedule of Attitudes toward Hastened Death (SAHD) (validated measure) Also measured Desire for Death Rating Scale (DDRS) (but only for validation of SAHD)	Exposure ✔ DHD outcome ✔	**X**	Spearman correlation coefficients	No significant correlation between social support and wish to hasten death (*r* = -0.06; non-significant)	Low risk of bias
Rosenfeld et al. (2006) [84] USA (New York State)	*N* = 372; mean age 44; 25% female; majority were Catholic and had less than a high school education; mixed ethnicity. Religiosity was measured but not reported	Cross-sectional study of patients with advanced AIDS recruited from nursing facilities and medical centres in New York City	Social support measured using the Duke University of North Carolina Functional Support Questionnaire (FSSQ) (validated measure)	Desire for hastened death (DHD) captured using two measures; Schedule of Attitudes toward Hastened Death (SAHD) (validated measure) Desire for Death Rating Scale (DDRS) (unvalidated measure) Scores on each measure were highly correlated but it was unclear which measure was used in the main analyses to create a binary measure of low *versus* high DHD	Exposure ✔ DHD outcomes ✔ and **X**	**X**	Correlations to identify predictors of DHD; variables identified as significant on univariate analysis were entered into a stepwise logistic regression model to identify the most parsimonious set of predictors (multivariable analysis)	Social support was significantly correlated with DHD ( *r* = –0.26 (*p* = 0.0001) but social support did not make a unique contribution to a model testing the association between a range of independent variables (sociodemographic and clinical) and DHD once depression and hopelessness taken into account (only these two variables provided significant unique contributions)	Low risk of bias
Rosenfeld et al. (2014) [85] USA (New York State)	*N* = 128; mean age 66; 52% female; majority were Catholic; mean years of education were 13.3 years; mixed ethnicity	Longitudinal study of patients with terminal cancer recruited shortly after admission to a palliative care hospital, questionnaire delivered at 2 time-points: T1 = baseline, T2 = 2–4 weeks later (originally a third data collection point was planned during this 42 month study but data were combined with T2 due to high attrition as many patients were too ill to complete the second or third assessment)	Social support measured using the Duke University of North Carolina Functional Support Questionnaire (FSSQ) (validated measure)	Desire for hastened death (DHD) measured using the Schedule of Attitudes Towards Hastened Death (SAHD) (validated measure)	Exposure ✔ DHD outcome ✔	**X**	Univariate analyses (ANOVA) to describe association between social support and predictors of low *versus* high DHD at baseline; variables found to be significant were entered into a stepwise logistic regression model to predict membership of four DHD trajectories (low DHD; rising DHD; falling DHD; high DHD) (multivariable analysis)	No association between mean social support score and the four trajectories of DHD (F = 1.12; p = 0.34) so not entered into final model	Low risk of bias
Schroepfer (2008) [62] USA (Michigan)	*N* = 96; mean age 74; 56% female; majority were Protestant, married, and had low religiosity; mean years in education was 12.1; mixed ethnicity	Cross-sectional interview study of terminally ill patients recruited purposively from palliative care hospitals and outpatient clinics	Social support measured using a binary measure of perceived support derived from discussion with patients (unvalidated measure)	Desire for hastened death (DHD) captured using a binary measure derived from asking if patients were considering hastening their death “Have you ever given serious thought to hastening the end of your life in any way?” (unvalidated measure)	Exposure **X** DHD outcome **X**	✔	Bivariate associations between DHD and predictors; Logistic regression, adjusted for years of education, religiosity, depression, pain intensity, marital status, parental status, direct social control (relating to support from caregivers to safeguard health), and social support (multivariable analysis)	Bivariate associations showed that conflictual social support was a significant predictor of DHD (*p* = < 0.001). In a fully adjusted model conflictual social support was significantly associated with DHD (adjusted OR = 25.46; *p*-value < 0.05)	High risk of bias purposive rather than representative sampling; unvalidated measures

Footnotes to all tables: *AIDS* Acquired Immune Deficiency Syndrome, *ALS* Amyotrophic Lateral Sclerosis, *BCSSS* Bottomley Cancer Social Support Scale, *COPE* Coping Orientations to Problems Experienced Scale, *DHD* Desire for Hastened Death; EAS = Euthanasia and Assisted Suicide, *HIV* Human Immunodeficiency Virus, *INQ-10* Interpersonal Needs Questionnaire, *MOS-SSS* Medical Outcomes Survey—Social Support Scale, *MS* Multiple Sclerosis, *SAHD* Schedule of Attitudes toward Hastened Death, *PAD* Physician-Assisted Death, *PAS* Physician-Assisted Suicide, *SCFS* Social and Family Contact Scale, *SES* Socioeconomic status, *SOS-V* State of Suffering V, *SSQ* Social Support Questionnaire, *UCLA* University of California Los Angeles, *QDD* Quality of Death and Dying Questionnaire

The majority of studies were cross-sectional (76%; 28/37). Of the 24% (9/37) that used longitudinal approaches, one involved repeated cross-sectional surveys of three consecutive representative samples of older adults, exploring societal changes in attitudes [63]. Across all included studies, the gender distribution was 50% female, and the mean age was 63 years. Included studies sampled populations in Australia (*n* = 2), Austria (*n* = 1), Canada (*n* = 4), France (*n *= 1), Germany (*n* = 2), the Netherlands (*n* = 3), Switzerland (*n* = 1), New Zealand (*n* = 1), the United Kingdom (UK) (*n* = 1), and selected states in the United States of American (USA) (*n* = 24). Figures do not add to 37 as some studies were conducted across multiple locations.

We ascertained whether EAS was legal in the country/state of sampling for 13/27 (89%) of studies, whilst acknowledging that people do travel abroad to enact EAS. Those sampling in countries where EAS was legal were set in Germany, the Netherlands, Switzerland, Canada, and selected US states (Oregon, Washington). The remaining four studies were conducted across multiple locations in which EAS legislation differed [66, 67, 69, 75].

The majority of studies surveyed patients with terminal illnesses or who were already in the process of accessing EAS (*n* = 31). Other studies, primarily assessing attitudes to EAS, sampled people not defined by a physical health condition or by a request for EAS (*n* = 6). Recruitment methods included: newsletter advertisements; direct recruitment from medical centres, euthanasia organisations, palliative care units or research registers; and referrals from healthcare professionals. Data were collected using survey methods (relatives; clinical staff; patients, with or without carer support), by interview or questionnaire; as well as using data from EAS application forms; and routine data from clinical or audit records within services. Sample sizes ranged from *n* = 6 to *n* = 7,534.

#### Exposure and outcome measurement

Overall, only 16% (6/37) of studies used validated measures for both exposure and outcomes, and 24% (9/37) used unvalidated measures for both (Tables 1– 3). The most common pattern was for use of a validated exposure measure with an unvalidated outcome measure (38%; 14/37), and 14% (5/37) used an unvalidated exposure measure and a validated outcomes measure. Three studies (8%) used a mixture of validated and unvalidated measures for either exposure or outcome. These methodological issues influenced judgements over study quality (Table 4).

**Table 4:** Quality appraisal of included studies (*n *= 37)

Citation	Positive association ✔ or **X**	Sampling approach (score range: 0–1)	Confounding variables (score range: 0–2)	Response rate (score range: 0–2)	Validity of social connectedness measure (score range: 0–2)	Validity of outcome measure (score range: 0–2)	Total score Total score divided by total maximum score (9)	Risk of bias
Achille & Ogloff (2004) [68]	✔	0	0	2 (77%)	2	1	56%	Medium
Arnold (2004) [69]	✔	0	1	0	2	0	33%	High
Berkman et al. (1999) [67]	✔	1	0	1 (34–36%)	2	0	44%	Medium
Blank et al. (2001) [70]	**X**	0	1	1 (48%)	0	0	22%	High
Breitbart et al. (1996) [71]	**X**	0	1	0	2	1	44%	Medium
Breitbart et al. (2000) [22]	✔	0	2	1 (22%)	2	2	78%	Low
Buiting et al. (2012) [63]	**X**	1	0	2 (69%)	2	0	56%	Medium
Cheung et al. 2020 [78]	**X**	1	0	1 (100%; analysis of routine clinical records)	1	0	33%	High
Chochinov (1995) [79]	**X**	0	1	1 (23%)	0	2	44%	Medium
Cicirelli (1997) [64]	**X**	0	2	0	2	0	44%	Medium
Comby & Filbet (2005) [56]	**X**	0	0	1 (100% but very small sample)	0	1	22%	High
Emanuel et al. (2000) [13]	**X**	0	0	2 (87%)	2	0	44%	Medium
Ganzini et al. (1998) [72]	**X**	0	0	2 (71%)	2	0	44%	Medium
Ganzini et al. (2006) [57]	**X**	0	0	1 (44%)	2	0	33%	High
Ganzini et al. (2008) [58]	**X**	0	0	1 (38%)	0	2	33%	High
Ganzini et al. (2009) [59]	**X**	0	0	1 (31%)	0	0	11%	High
Himchak (1997) [73]	**X**	0	2	0	2	1	56%	Medium
Kelly et al. (2003) [80]	✔	0	2	1 (49%)	2	1	67%	Low
Lulé et al. (2014) [74]	**X**	0	1	0	2	2	56%	Medium
Marrie et al. (2017) [75]	**X**	0	1	2 (70%)	2	0	56%	Medium
O’Mahony (2005) [23]	✔	0	0	1 (49%)	2	2	56%	Medium
O’Mahony (2010) [81]	✔	0	1	1 (49%)	2	1	56%	Medium
Pacheco et al. (2003) [76]	**X**	0	0	2 (79%)	1	0	33%	High
Rodin et al. (2007) [82]	**X**	1	2	1 (59%)	2	2	89%	Low
Rosenfeld et al. (2000) [83]	**X**	0	0	2 (60%)	2	2	67%	Low
Rosenfeld et al. (2006) [84]	**X**	1	2	2 (87%)	2	2	100%	Low
Rosenfeld et al. (2014) [85]	**X**	0	2	1 (13%)	2	2	78% (7/9)	Low
Ruijs et al. (2014) [53]	**X**	0	0	1 (51%)	1	2	44%	Medium
Schroepfer (2008) [62]	✔	0	2	0	0	0	22%	High
Seidlitz et al. (1995) [66]	**X**	1	1	0	0	0	22%	High
Smith et al. (2011) [52]	**X**	0	0	1 (38%)	1	2	44%	Medium
Smith et al. (2015) [60]	**X**	0	0	0	2	2	44%	Medium
Snijdewind et al. (2015) [11]	✔	1	0	2 (91%)	0	2	56%	Medium
Stolz et al. (2017) [65]	✔ and **X**	1	2	2 (85%)	1	0	67%	Low
Stutzki et al. (2014) [77]	✔	1	0	1 (50%)	0	0	22%	High
Virik & Glare (2002) [55]	**X**	0	0	1 (100% but very small sample size)	0	2	33%	High
Wilson et al. (2007) [61]	✔	1	0	1 (41%)	1	0	33%	High

Footnote: Grading of the adapted QATSO scores was as follows: 0% -33% = low quality i.e., high risk of bias; 34%- 66% = medium quality i.e., medium risk of bias; 67% -100% = high quality i.e., low risk of bias

### Exposure measurement

To measure exposure, 24/37 studies (65%) used validated measures of social connectedness. The majority of studies (68%, 25/37) measured social support, whilst 11% (4/37) measured loneliness, and 19% (7/37) used other measures (i.e., social isolation, thwarted belongingness, satisfaction with relationships). Measures used to capture social support included the Social Support Questionnaire (SSQ) [86], the Social Networks in Adult Life Survey [87], the Duke University of North Carolina Functional Social Support Questionnaire (FSSQ) [88], the Medical Outcomes Study – Social Support Survey (MOS-SSS) [89], the Family Relationships Index (FRI) [90], the Coping Orientations to Problems Experienced Scale (COPE) [91], and the Multidimensional Scale of Perceived Social Support (MSPSS) [92]. Measures used to capture loneliness included the De Jong Gierveld Loneliness Scale [93] and the UCLA Loneliness Scale [94]. Some studies used instruments capturing social connectedness that had been specifically validated in palliative care populations, such as the State-of-Suffering V (SOS-V) [54], whilst others used specific items from validated measures e.g., the Quality of Death and Dying Questionnaire (QODD) [95].

### Outcome measurement

Studies measured outcomes in three main ways:

Requested/actual EAS; measured using routine clinical records, such as GP, palliative care or physician records, or registers of those contacting palliative care services or voluntary sector organisations (representing those with terminal illness or advocating for EAS) seeking information on how to make a request. Death by EAS was measured objectively using records in hospices, medical centres, and voluntary sector organisations registering deaths by EAS. Four studies captured request for EAS [53, 55, 56, 60]. A further four studies included not only requests but also cases where a request for EAS had ended in death by EAS [11, 52, 58]. One study involved patients ranking lack of support in relation to other reasons for having requested PAS [59].

Attitudes towards EAS; captured using a range of unvalidated measures, including using standardised questions taken from national surveys or from previous research studies, justified based on face validity. Agreement with these measures was elicited from patients using questionnaires or interviews. In some cases, attitudes to EAS were elicited from caregivers to capture their views on EAS, whether they would support or oppose the patient’s decision to seek EAS, the perceived likelihood that the patient would request EAS and whether the patient had discussed EAS with them or hoarded drugs. Seventeen studies captured attitudes towards EAS [13, 59, 61, 63–76].

Desire for hastened death (DHD); measured using the Schedule of Attitudes Towards Hastened Death (SAHD) [83], or the Desire for Death Rating Scale (DDRS) [79], and in some studies using unvalidated measures of DHD. The SAHD is a self-report inventory and has been validated formally [83, 96]. The DDRS is a clinician-rated measure and constitutes a set of questions created for a study published in 1995 [79], since used widely as a set of standardised questions, but not formally validated. Nevertheless, the DDRS is highly correlated with the SAHD [83]. Fifteen studies captured DHD [22, 23, 57, 59, 62, 74, 77–85].

### Methodological quality

We judged 19% (7/37) of studies to be at low risk of bias, 46% (17/37) at medium risk, and 35% (13/37) at high risk (Table 4). The main limitations identified included use of purposive or opportunistic sampling and/or unvalidated measures, lack of adjustment for key confounders, and uncorrected multiple testing. Only ten studies (27%) used representative sampling, and 21 (57%) used multivariable regression modelling. The majority (29/37; 78%) reported their response rate. This varied substantially depending on the methodology used (median response rate = 50%, IQR = 38%-79%).

### Association between social connectedness measures requested/actual EAS (*n* = 9)

Overall, nine studies investigated associations between social connectedness and requested/actual EAS, of which four were rated as having a medium risk of bias and five at a high risk of bias. Focussing on those rated at medium risk of bias, one Dutch study of applications to an end-of-life clinic reported an association between loneliness and a request for EAS being rejected, although loneliness was one of at least 20 sociodemographic and clinical variables tested (Snijdewind et al., 2015) [11]. All other studies reported the absence of a significant association. A study in a Dutch sample of patients with terminal cancer found no associations between loneliness, unsatisfactory social contacts, insufficient support or subjective isolation and a request for EAS (Ruijs et al., 2014) [53]). A US study of patients with terminal illness recruited from end-of-life advocacy organisations and hospices in Oregon State found no association between social support and a request for EAS (Smith et al., 2015) [60]). A US study of the relatives of patients in Oregon State with terminal illness recruited from health services, amyotrophic lateral sclerosis organisations, and euthanasia advocacy organisations found no association between three social connectedness measures and requested/actual EAS (Smith et al. (2011) [52]. Note that EAS was legal in all the above settings at the time of sampling. Together these studies do not support an association between social connectedness and requested or actual EAS due to the potential for Type I error in the only study reporting an association with loneliness, as well as other methodological issues.

Our GRADE rating denoted low certainty of this evidence for the outcome of requested/actual EAS.

### Association between social connectedness measures and attitudes to EAS (*n* = 16)

Overall, 16 studies investigated associations between social connectedness and attitudes to EAS, of which one was rated as having a low risk of bias, ten at a medium risk of bias, and five at a high risk of bias.

Focussing on the only study rated as low risk of bias, which sampled Austrian care-dependent adults, this reported an association between a crude measure of social isolation (living alone) and reported approval of availability of euthanasia as well as of hypothetical utilisation of euthanasia but it was not associated with approval of availability of assisted suicide or hypothetical utilisation of assisted suicide (Stolz et al., 2017) [65]. Thwarted belongingness was not associated with any of these four outcomes (Stolz et al., 2017) [65]. Whilst this study was rated as good quality methodologically, there was a clear issue of generalisability in that those sampled were community-dwelling care-dependent adults over 50 years of age, defined by having a physical or mental disability that was expected to last at least 6 months. In not being defined by a life-limiting illness, their attitudes to EAS were elicited in a situation in which many would not be considering EAS. Additionally, EAS was illegal in Austria at that time.

Focussing on the ten studies in the studies rated at medium risk of bias, in a US study of patients with multiple sclerosis in the states of Oregon (where EAS was legal) and Michigan (where EAS was illegal), poor social support was negatively significantly associated with having ever thought about assisted suicide but there was no evidence to support an association with social isolation (Berkman et al., 1999) [67]. However, in a US-wide study of patients with multiple sclerosis, there was no association between perceived social support and attitudes to EAS Marrie et al. (2017) [75]. In a study of patients with amyotrophic lateral sclerosis in Canada, the US, and the UK (in the context of EAS being legal in Canada and two US states) there was a significant negative correlation between availability of social support and whether patients were contemplating EAS (Achille & Ogloff, 2004) [68]). However, a German study of patients with amyotrophic lateral sclerosis found no association between perceived social support and attitudes to EAS (Lulé et al., 2014) [74], and a US study sampling patients with amyotrophic lateral sclerosis in Oregon and Washington found no significant differences in social support between patients who would and would not consider EAS (Ganzini et al., 1998) [72]. In both studies EAS was legal for those sampled. In a US-wide study of patients with terminal illness no association was found between social support and whether patients had discussed EAS or hoarded drugs (Emanuel et al. (2000) [13]. In a US study of patients in New York state with HIV, there was a significant association between two dimensions of social support and attitude to EAS (Breitbart et al. (1996) [71]. However, this had issues of generalisability because attitudes to death among HIV-infected patients sampled in the mid-1990s would have been conditioned by the public understanding of HIV prognosis at that time.

A number of studies in the medium risk of bias category sampled people who were not defined by life-limiting illness. This means that findings (all found no evidence to support an association) would not be generalisable to those with terminal illness. One Dutch study of older adults (not defined by life-limiting illness) found no association between loneliness and attitudes to EAS (Buiting et al., 2012) [63]. A US study sampling older adults in Indiana state (also not defined by life-limiting illness) found no association between loneliness or perceived social support and attitudes to EAS (Cicirelli, 1997) [64]. Similarly, a US study sampling older adults (with no apparent life-limiting illness) in New Jersey, New York, and Pennsylvania states found no association found between social support and attitudes to EAS (Himchak, 1997) [73].

Together these studies did not provide good evidence to support an association between social connectedness and attitudes to EAS given issues of generalisability, methodology, and the general issue of the validity of attitude measurement as a proxy for EAS probability.

Our GRADE rating denoted very low certainty of this evidence for the outcome of attitudes to EAS.

### Association between social connectedness measures and desire for hastened death (*n* = 14)

Overall, 14 studies investigated associations between social connectedness and DHD, of which six were rated as having a low risk of bias, four at a medium risk of bias, and four at a high risk of bias.

Most of the six studies rated at low risk of bias related to patients with advanced cancer. In an Australian study of patients with terminal cancer, the three dimensions of social support investigated (family cohesion; number of social supports, satisfaction with social supports) were associated with DHD (Kelly et al., 2003) [80]. However, in three US studies of patients with terminal cancer in New York state there was no evidence to support a significant association between perceived social support and DHD (Breitbart et al., 2000) [22] and no association found between social support and DHD (Rosenfeld et al., 2000) [83] (Rosenfeld et al., 2014) [85]. Similarly, in a Canadian study of patients with metastatic cancer, social support was not associated with DHD (Rodin et al. (2007) [82]). Finally, in a US study sampling patients with advanced AIDS in New York State, social support was not associated with DHD (Rosenfeld et al. (2006) [84]. Note that of these studies, EAS was legal only in Canada.

Of the four studies rated at medium risk of bias, two were US analyses of the same sample of patients with terminal cancer in New York State, which presented weak evidence to support an association between poor social support and DHD (O’Mahony et al., 2005) [23] (O’Mahony et al., 2010) [81], and weak evidence to support an association between living alone and DHD (O’Mahony et al., 2005) [23]. The other two studies in this category reported no significant association. A German study of patients with amyotrophic lateral sclerosis found no association between perceived social support and DHD (Lulé et al. (2014) [74]. In a Canadian study of patients with terminal illness in hospital palliative care units there was no association between perceived social support and DHD (Chochinov et al., 1995) [79]. Of these studies, EAS was legal only in Germany and Canada.

Together these studies, did not provide good evidence to support an association between social connectedness and DHD given contradictory findings and issues of methodology. DHD is also a poor proxy for EAS probability, with issues over comparability of measurement in areas where EAS is and is not legal.

Our GRADE rating denoted moderate certainty of this evidence for the outcome of DHD.

## Discussion

### Main findings

Our review identified a body of literature that investigated the associations between a broad range of social connectedness measures and those capturing actual or requested EAS (our main research question), as well as those more distally related to it (attitudes to EAS; DHD). Generally, there was no evidence to support an association between different constructs of social connectedness and requested or actual EAS, and very little evidence to support an association with attitudes to EAS or an association with DHD. The quality of studies was poor, with 62% rated as medium/high risk of bias, with GRADE ratings indicating that the certainty of recommendations varied from very low to moderate. No studies related specifically to psychiatric euthanasia, which is striking given the high prevalence of loneliness in such cases [13]. No studies related to EAS in the context of intellectual disability and/or autism, in which the prevalence of loneliness is also high [97]. It is possible that the nature of any associations between social connectedness and EAS in the context of severe psychiatric disorder, intellectual disability and/or autism will differ from those in the context of terminal physical illness.

Comparing our findings to a previous systematic review focussed on older adults [46], both their review and ours concluded that there was no or little evidence to support an association between loneliness or low satisfaction with family relationships with request for or attitudes to EAS. In our review, hopelessness and depression were risk factors supported by more consistent evidence for an association with DHD and attitudes and EAS, even though this was not an inclusion criterion. The seven studies rated at low risk of bias in this review (albeit all cross-sectional) identified hopelessness and/or depression to be associated with DHD [22, 80, 82–84], or with attitudes to EAS [75], apart from one, which found no association of depressed affect with positive attitudes to EAS [65].

### Strengths and limitations

To our knowledge, this is the first systematic review of published studies describing the association between social connectedness and measures relating to EAS across all age groups, using a broad range of concepts of social connectedness and EAS-related outcomes. Data extraction was conducted and reviewed independently by four authors, achieving a high level of rigor in distilling key findings and characteristics. Use of the GRADE framework to evaluate the strength of evidence for each outcome added to the robustness of our conclusions. Clear sub-categorisation was used to address substantial heterogeneity across exposure/outcome measures. We used an accepted quality appraisal tool, the (QATSO) [49], to delineate the limitations of the current evidence base. Our synthesis of findings focussed on higher quality studies, so that conclusions were based on those with lower risk of bias. We acknowledged that participants who had requested EAS (rather than enacted EAS) may not be representative of those who die by EAS, and that DHD may not correlate well with enacted EAS, which is why these outcomes were grouped separately.

The main limitation of our review is that the majority of studies included in the review had methodological limitations, mainly due to unvalidated exposure/outcome measures, cross-sectional design, unadjusted analyses, and multiple testing. Some studies sampled populations without a life-limiting illness, or in countries where EAS was illegal, or included relatives’ views. None of the longitudinal studies had sufficient statistical power to analyse fatal outcomes, and any fatal outcomes were included within combined measures of requests (enacted and non-enacted) for EAS. However, conducting larger longitudinal studies may not add much to knowledge given the difficulties of following up patients who had made EAS requests longitudinally. Finally, our strategy did not include identification of grey literature, and there also remains the possibility of publication bias in under-representing unpublished studies reporting no statistically significant associations [98].

### Clinical, policy and research implications

The lack of evidence to support an association between social connectedness and requested/actual EAS and related measures suggests that other factors may dominate end-of-life decision-making processes. Patients who seek EAS experience intolerable suffering that cannot be relieved. Addressing potentially modifiable factors such as loneliness and poor perceived social support would improve quality of life, as would addressing depression and hopelessness (which are clinical factors likely to make a more substantial contribution to requests for EAS). Given the contribution of loneliness to depression in older people [99] and the bidirectional association between social disconnectedness and poor mental health [100, 101], loneliness and depression are likely to be closely interlinked. Whilst depression (and hopelessness, as a symptom of depression) are modifiable using evidence-based treatments, loneliness is also potentially modifiable, whether in general population samples [102] or among those with pre-existing mental illness [103]. This is a growing research field, with great interest in the development and evaluation of interventions to address loneliness [102–104]. Intervening in this way might be one means of preventing the onset of depression that might otherwise lead to DHD and/or EAS.

The reasons given by patients for requesting EAS, including perceived poor quality of life and fear of future suffering, might be allayed by Advance Care Planning; a process for discussing their preferences and priorities for their future care, including situations in which they might refuse treatment (or request EAS, where legal). In the context of serious physical illness Advance Care Planning should start by checking mental capacity and follow local clinical guidelines [105]. In the context of psychiatric disorder, Advance Care Planning can include discussions about psychiatric admission, use of electroconvulsive therapy, and refusal of treatment, but there is a comparative lack of clinical guidelines to inform this process [106]. Family involvement is very important in this process [107], but for patients who feel less socially connected this may be problematic.

Given limited support for an association between social connectedness and EAS, the rationale for inquiring about social connectedness in patient populations who might consider EAS is therefore that intervening is likely to improve quality of life. Clinical guidance on the assessment and management of patients with terminal illness, severe physical disability and severe or enduring mental distress, including for those who request EAS, should therefore include inquiring about loneliness, isolation and social contacts alongside standard questions about pain and untreated mental or physical health problems. Information on social connectedness would provide valuable opportunities for clinical and voluntary services serving such populations to consider what support might be indicated to reduce distress.

In view of the methodological limitations of many included studies, we recommend that future research studies employ representative sampling and use of validated measures and appropriate statistical models. We also acknowledge the limited applicability of existing theoretical models of suicide to the phenomenon of EAS and note the need for a conceptual review to inform appropriate iterations of such models.

## Conclusions

This review found no evidence to support an association between social connectedness and requested/actual EAS, and weak evidence to support an association between social connectedness and related measures (attitudes to EAS; desire for hastened death). The strength of the evidence was generally low, mainly due to high risk of bias, and there is a need for further high-quality research investigating these associations. Nevertheless, modifying any distressing aspects of poor social connectedness could improve quality of life in those who experience intolerable suffering due to physical or mental illness that cannot be relieved. The evidence base for interventions to address loneliness and social isolation is growing, and more work is needed to develop and evaluate effective interventions to target these in a range of settings, including end-of-life care.

### Supplementary Information

**Supplementary Material 1. **

**Supplementary Material 2. **

**Supplementary Material 3. **

## Data Availability

Data sharing is not applicable to this article as no datasets were generated or analysed during the current study. Full citations are provided for all included studies.
