# Supplementary material for: The association between social connectedness and euthanasia and assisted suicide and related constructs: systematic review
Source: BMC Public Health. 2024 Apr 16;24:1057. doi: 10.1186/s12889-024-18528-4 (PMC11020194; doi:10.1186/s12889-024-18528-4)
Supplement: Supplementary file 3 — Supplementary Material 3. [file 12889_2024_18528_MOESM3_ESM.docx]

**Appendix 2: Scoring of quality assessment criteria**

Total score: 0-9

| **Score** | **Sampling approach** | **Confounding variables** | **Response rate** | **Validity of social connectedness measure** | **Validity of outcome measure** |
| --- | --- | --- | --- | --- | --- |
| 0 | Opportunistic sampling | No attempt to control for confounding variables | Response rate not reported | Single item assessment with no valid or reliable backing | Unclear assessment of DHD/EAS  Measure is invalid or unreliable |
| 1 | Representative samples | Some attempt to control for confounding variables e.g. demographics | Participant response rate less than 60% | 1 or 2 items taken from a standardised measure of a wider psychological assessment | 1 or 2 items taken from a standardised measure of a wider psychological assessment to assess thoughts about EAS |
| 2 | n/a  (max score for this criterion is 1) | Accounts for additional confounding variables e.g. history of mental health problems, other psychological variables | Participant response rate more than 60% | Full measure or subscale targeted to explore loneliness | Full measure or subscale targeted to assess DHD/EAS  Hospital records, formal requests |

**Scoring method**: Total score divided by total number of all applicable items:

0-33% = high risk of bias

34-66% = medium risk of bias

67-100% = low risk of bias

**Appendix 3: GRADE scoring criteria for studies investigating the association between social connectedness and EAS**

Each GRADE domain could receive ‘no concerns’, ‘borderline’ or ‘serious concerns’ rating. The overall certainty for each outcome started as high and was downgraded for any ‘serious concerns’. The GRADE domains were operationalized in the following way:

1. Study quality: ‘Serious concerns’ were noted if ≥ 50% of the contributing studies were of low quality and ‘no concern’ if > 50% were of high quality based on quality ratings (see Table 4)
2. Inconsistency: Consistency of the direction of change and the magnitude of effects across the research evidence was evaluated. ‘No concerns’ were noted when most studies reported associations in the same direction, or where there was only one contributing study and therefore the inconsistency was impossible to tell. ‘Serious concerns’ were noted when there was evidence of opposite directions of association.
3. Indirectness: A judgement was made on the degree of similarity of the research evidence with the research question of interest, reflecting on how directly the available evidence answered the specific research questions set out in the review. For example, unvalidated outcome measures and those that were deemed less directly related to a research question contributed to down-ratings for this domain. ‘Serious concerns’ were also raised when most contributing studies included a population with no life-limiting illness instead of a physical or mental illness causing intolerable suffering that cannot be relieved.
4. Imprecision: A judgement was made based on the total number of contributing studies and their sample size. The sample size threshold used for the relevant analysis was >100 for each outcome. ‘Serious concerns’ were noted when there was only one contributing study, regardless of its sample size.
5. Publication bias: We considered if studies contributing to an outcome reported significant and non-significant results, or if publication bias was likely due to missing evidence. If the only studies identified as eligible in the search reported positive results then the rating was downgraded (if not already at lowest rating).

GRADE scoring results:

| **Outcome: requested/actual EAS**  Result: Low certainty  Contributing studies: 9 - see Table 1 | | |
| --- | --- | --- |
| Domain | Assessment | Outcome |
| Study quality/risk of bias | Serious concerns:  no studies rated as low risk of bias, four were rated as having a medium risk of bias and five at a high risk of bias. | Downgraded to moderate certainty. |
| Inconsistency | No concerns:  studies were consistent in their results in that 8 of 9 showed negative findings, and the only study with positive findings was exploratory in nature, raising the question of Type I error. | No change. |
| Indirectness | No concerns:  all studies investigated samples with terminal illness in areas where EAS was legal. | No change. |
| Precision | Borderline concerns:  sample sizes ranged from n=6 to n=645 participants. | No change. |
| Publication bias | Serious concerns:  Small number of studies.  The only study with positive findings tested loneliness among at least 20 sociodemographic and clinical variables. | Downgraded to low certainty. |

| **Outcome: attitudes to EAS**  Result: Very low certainty  Contributing studies: 16 - see Table 2 | | |
| --- | --- | --- |
| Domain | Assessment | Outcome |
| Study quality/risk of bias | Serious concerns:  only one study rated as low risk of bias, ten were rated as having a medium risk of bias and five at a high risk of bias. | Downgraded to moderate certainty. |
| Inconsistency | Serious concerns:  many studies in similar populations had conflicting findings. | Downgraded to low certainty. |
| Indirectness | Serious concerns:  many studies investigated samples with no life-limiting illness; many studies sampled in areas where EAS was illegal. | Downgraded to very low certainty. |
| Precision | Borderline concerns:  sample sizes ranged from n=24 to n=7,534 participants. | No change as rating already very low and no justification to upgrade. |
| Publication bias | No concerns:  Relatively small number of studies.  Balance of positive and negative findings. | No change as rating already very low and no justification to upgrade. |

| **Outcome: DHD**  Result: Moderate certainty  Contributing studies: 14 - see Table 3 | | |
| --- | --- | --- |
| Domain | Assessment | Outcome |
| Study quality/risk of bias | Borderline concerns:  six studies were rated as low risk of bias, four were rated as having a medium risk of bias and four at a high risk of bias. | No change. |
| Inconsistency | Serious concerns:  many studies in similar populations had conflicting findings. | Downgraded to moderate certainty. |
| Indirectness | Borderline concerns: half of studies used unvalidated measures of DHD; all studies investigated samples with life-limiting illness. | No change. |
| Precision | No concerns:  sample sizes ranged from n=64 to n=771 participants. | No change. |
| Publication bias | No concerns:  Relatively small number of studies, but balance of positive and negative findings. | No change. |
